# Exploration of whether socioeconomic factors affect the results of priority setting partnerships: updating the top 10 research priorities for the management of Parkinson’s in an international setting

**DOI:** 10.1136/bmjopen-2021-049530

**Published:** 2022-06-29

**Authors:** Francesca Bowring, Jessica Welch, Charlotte Woodward, Christine Lo, Michael Lawton, Patricia Sulzer, Anne-Marie Hanff, Rejko Kruger, Inga Liepelt-Scarfone, Michele T Hu

**Affiliations:** 1 Department of Clinical Neurosciences, University of Oxford Nuffield, Oxford, UK; 2 Division of Surgery and Interventional Science, University College London, London, UK; 3 Oxford University Hospitals NHS Foundation Trust, Oxford, UK; 4 Biostatistics, University of Bristol, Bristol, UK; 5 Eberhard Karls University Tubingen Hertie Institute for Clinical Brain Research, Tubingen, Germany; 6 Transversal Translational Medicine, Luxembourg Institute of Health, Strassen, Luxembourg; 7 Faculty of Science, Technology and Medicine, University of Luxembourg, Esch-sur-Alzette, Luxembourg; 8 Department of Epidemiology, Maastricht University Medical Centre+, Maastricht, The Netherlands; 9 Translational Neuroscience, Université du Luxembourg Luxembourg Centre for Systems Biomedicine, Esch-sur-Alzette, Luxembourg; 10 Parkinson Research Clinic, Centre Hospitalier de Luxembourg, Strassen, Luxembourg; 11 Nuffield Department of Clinical Neurosciences, University of Oxford, Oxford, UK

**Keywords:** parkinson-s disease, statistics & research methods, qualitative research, neurology

## Abstract

**Abstract:**

**Objectives:**

Explore whether socioeconomic differences of patients affect the prioritisation of pre-existing research questions and explore the agreement between healthcare professionals (HCP) and patients in priority setting partnerships (PSPs).

**Design and setting:**

Prospective, three centre survey across UK (400 participants), Tuebingen (176 participants) and Luxembourg (303 participants). People with Parkinson’s (PwP), research participants, relatives and HCP associated with three Parkinson’s cohort studies were invited to participate, along with linked centres (clinical care settings, research groups, charities). Responders were encouraged to pass on the survey to friends/families/carers.

**Methods:**

The survey involved rating the importance of research questions on a Likert scale, allowing for the generation of one new question participants felt was particularly important. Collection of demographic information allowed for comparisons of priorities across a range of socioeconomic variables; the top 10 research priorities for each group were then compared. Questions added by participants were subject to a thematic analysis.

**Results:**

879 participants completed the survey (58% PwP, 22% family/friends, 13% HCP, 4% carers). Finding the best form of physiotherapy for PwP was the number one priority across the majority of analyses. HCP were the only subgroup not to place physiotherapy in the top 10. Factors most likely to affect prioritisation in PwP included educational level, presence of carer support and disease duration. There was little difference between other socioeconomic categories.

**Conclusions:**

Socioeconomic factors modestly influenced some research priority ratings but did not significantly affect the top priority in most comparisons. Future studies must ensure patients from a range of socioeconomic backgrounds are recruited, ensuring results generalisable to the public while also identifying any key disparities in prioritisation. PSP should also take care that HCP do not skew results during prioritisation of questions, as in this study the most important priority to patients was not identified by professionals.

**Strengths and limitations of this study**

- This is the first priority setting partnership (PSP) to explore the influence of socioeconomic factors on the prioritisation of research questions, exploring their role across several European Parkinson’s centres.
- A large sample of 879 participants completed the survey to rate the priority questions.
- Despite best efforts, there were limited responses from ethnic minorities and from people supported by carers or in care/nursing homes.
- Conducted only in Western Europe, therefore, may not be generalisable to an international audience.

## Introduction

 Priority setting partnerships (PSPs) (online supplemental material table S1) aim to make research more meaningful to patients by ascertaining the questions about medical conditions that are of the greatest importance to patients, their friends/family members and associated healthcare professionals (HCP).^1 2^ Developing questions with a focus on the effectiveness of treatment options and care provision important to patients could improve participation in clinical trials, better inform research funding strategies and improve healthcare policies for patients; instead of the influence from ‘big pharma’.^3^,^5^ It has not previously been explored how socioeconomic factors affect the prioritisation of these questions. PSP groups vary in methodology but normally follow a format whereby:^6 7^

1. ‘Research uncertainties’ (subjects not yet answered) are generated by stakeholders through survey.
2. Uncertainties are turned into questions following systematic search of research databases.
3. The identified questions are ranked in order during an interim survey to form a smaller list of questions.
4. The reduced list is then ranked by a steering group to produce the final top 10 priorities.

Parkinson’s is a neurodegenerative disorder (symptoms include rigidity and tremor), with depression, dementia and mild cognitive impairment being more prevalent in people with Parkinson’s (PwP).^8^,^14^ It has been hypothesised there are different subtypes of Parkinson’s, contributing to the heterogeneity of symptoms observed in the clinical setting.^1215^,^18^ This could be problematic when trying to achieve consensus in research priorities, as Parkinson’s experience can differ substantially. Furthermore, Parkinson’s priorities for research topics may change depending on confounders (eg, disease duration and socioeconomic factors).

In 2014, research priorities for the management of Parkinson’s were identified (online supplemental material figure S1) by a focus group led by Parkinson’s UK, comprising 27 participants after an interim ranking round with 475 participants.^19^ Results were subsequently presented at the 2016 Oxford Parkinson’s Disease Centre (OPDC) cohort participant open day where attendees were asked to rate the list; results differed significantly. We therefore decided to formally explore whether socioeconomic differences influenced research priorities within CENTRE-PD, a Horizon2020 project allowing University of Luxembourg (UL), University of Tübingen (EKUT) and University of Oxford (UOXF) to share expertise, synchronise research cohort protocols giving greater statistical strength to analyses and enable ease of replication of studies and processes.^20^ This study also aims to include more HCP; a limitation in the 2014 study.^19^ Fundamentally, the three centre cohort studies are longitudinal, observational studies following PwP, at-risk people and age-matched controls to better understand the pathological pathways of Parkinson’s.^21^,^25^ As the cohorts are based in different countries, geographical comparisons can be made to see if there are any cross-cultural differences, something previously not done.^19^

**Figure 1:**
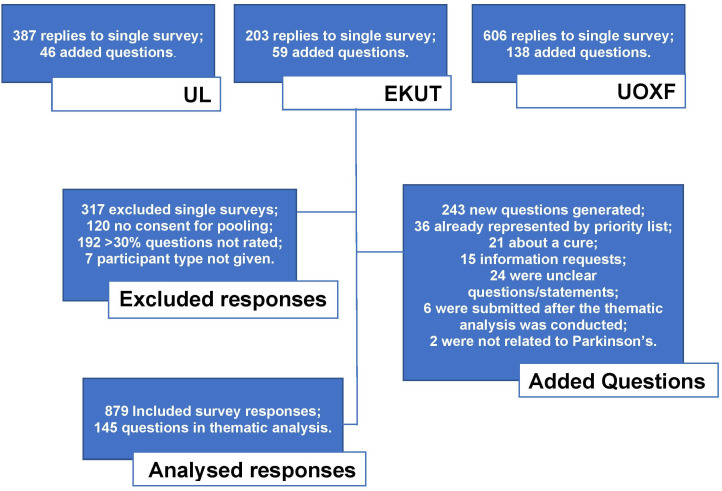
Study flow chart of responses (response summary, exclusions, added questions and final data for analysis). EKUT, University of Tübingen; UL, University of Luxembourg; UOXF, University of Oxford.

## Objectives

The primary aims of this study were to explore:

- Whether research priorities for the management of Parkinson’s change based on geographical location or other socioeconomic factors.
- Whether priorities of researchers/HCP align with that of PwP.

The secondary outcome from this was an updated research priorities list into Parkinson’s management, and provide guidance on future systematic review questions that are important to PwP.

## Methods

### Generation of questions

In the 2014 study,^19^ 94 questions were created for ranking using a typical PSP method. There was concern too many questions would make attention retention difficult for respondents, be too time consuming and reduce responses from our cohort participants. Therefore, the top 26 priorities (online supplemental material figure S2) from the interim ranking round were deemed sufficient. During the informal survey at the OPDC cohort open day, of the 147 responses, nine statements were included pertaining to speech difficulties. As this was of particular interest to our participants, we included this as a question in our survey. We also gave a free-text element option, allowing participants to write down a question they felt had been missed. Thus, the survey had 27 questions to rate, with an additional free-text question.

### Participant recruitment

Based on the median number of participants (n=386) (online supplemental material table S2) in JLA interim surveys, we aimed to recruit >400 people. There were no specific eligibility criteria as this was a feedback survey. All participants of the research cohorts associated with CENTRE-PD were invited to complete the survey once by post and/or email between July 2018 and January 2019. Cohort participants were invited to share the survey with friends/family as we were interested in the views of all people affected by Parkinson’s. Members of email lists associated with the cohorts, study centres or CENTRE-PD were invited, Parkinson’s UK also distributed the survey to interested persons. The rating pack and link for voluntary completion were sent by email to HCPs. Oxfordshire care homes were contacted twice to encourage responses from carers, nurses and PwP living there, (these people were identified as least likely to respond and likely to have lived with Parkinson’s for longer). The survey was published by local research outreach teams, the hospital trust website, in hospital staff bulletins, university department bulletins, on the OPDC website, the OPDC and trust social media accounts, and in the local National Institute for Health Research (NIHR) newsletter. In Luxembourg the German and French paper version of the rating pack was sent with the newsletter to 646 patients and 996 controls of the Luxembourg Parkinson’s study. PwP were invited to share the survey with friends/family. Additionally, about 700 HCPs receiving the newsletter were invited to complete the online version. Tuebingen contacted 243 people via email, as well as recruited participants at a patients’ day.

### Patient and public involvement

Patients, their partners, family and related HCP from three cohort studies across Europe were invited to complete a survey asking about their sociodemographic variables (online supplemental material tables S3–S5 and figures S3–S5) and to rank their priorities for research into Parkinson’s disease. Results of the study were disseminated via patient newsletter.

### Completing the survey

Parkinson’s UK were contacted for permission to adapt literature created during their PSP group and a rating pack was created. The survey and rating pack were translated at each site using the WHO process of translation and adaption of instruments.^26^ At the UOXF, the online version priority questions were presented in a random order (by the software) to reduce the risk of the same question(s) repeatedly being missed. The average, online survey completion time was 11 min (data from UOXF).

Surveys partially completed were assessed for inclusion or had the following imputations:

- If responder type was missing the survey was excluded.
- If demographic information was missing, ‘prefer not to say’ was selected.
- If 30% or more of the questions for rating were missed, the response was excluded.
- If fewer than 30% of the ratings were missed, the median score was imputed. There was a total of 118 imputed data points across all priority questions (0.5% of total ratings), with Q5, Would the monitoring of dopamine levels in the body (eg, with blood tests) be helpful in determining medication timing and amount (dose)? missed the most (n=10 missing data points).

### Analysis

To compare priorities between socioeconomic groups of PwP, we collected the following information:

- Participant type.
- Geographical location.
- Disease duration*.
- Gender*.
- Ethnicity (UOXF only)*.
- Education level*.
- Economic status*.
- Living arrangements*.

(online supplemental material figures S6–S14).

*For PwP analysis

To calculate the top 10 for each subgroup, questions were ordered by the percentage agreeing that it was a high priority (score 7–9) (online supplemental material tables S6–S13). Where this was not sufficient to produce the top 10, median, IQR and range of scores were included. In one analysis this was still insufficient and the number of participants who voted the priority as nine had to be used.

To compare agreement between subgroups of whether each of the 27 primary questions were in the top 10 or not for that subgroup, Cohen’s kappa (K) tests were run (online supplemental material tables S14–S22). Interpretation recommendations were used where: 0.41–0.60 was moderate agreement, 0.61–0.80 substantial, and 0.81–1.00 near perfect agreement.^27 28^ For comparison of the score distributions of each priority question, Mann-Whitney-Wilcoxon (MWW) or Kruskal-Wallis (KW) test was used, as required by the number of subgroups analysed.

For the thematic analysis of added questions, each question was associated with up to four themes (eg, ‘gut microbiota’) by two raters autonomously, before convening to establish agreement. Where necessary, a third team member gave input. Themes were then grouped and counted, and the ten most recurring themes were made into research questions.^29^

## Results

In total, 1196 people responded to the survey, of which 879 were included for analysis (figure 1 and table 1). In the pooled analysis to establish the top 10 research priorities for Parkinson’s, the joint first questions (79.1% agreement) were:

- Q19 ‘What is the best type and dose of exercise (physiotherapy) for improving muscle strength, flexibility, fitness, balance and function in people with Parkinson’s?’
- Q9 ‘What drug treatments are best for the different stages of Parkinson’s?’
  The CENTRE-PD top 10 is displayed in table 2; the descriptive statistics for all questions are presented in table 3 with added questions presented in table 4. Table 5 highlights the top 10 research priorities and the ranking, as rated by each subgroup for visual comparison.

**Table 1:** Percentage of responses by stakeholder group at each site

Cohort site	Stakeholder group		
	PwP (%)	HCP (%)	Other (%)
Luxembourg	38	24	38
Tubingen	90	4	6
UK	59	8	33

HCP, healthcare professional; PwP, people with Parkinson’s.

**Table 2:** The final top 10 research priorities from pooled analysis of all sites and participants

CENTRE-PD top 10 research priorities for Parkinson’s				
(n=879)				
Rank	Question no	Question	N who ranked ≥7	% Agreement
1*	19	What is the best type and dose of exercise (physiotherapy) for improving muscle strength, flexibility, fitness, balance and function in people with Parkinson’s?	695	79.1
1	9	What drug treatments are best for the different stages of Parkinson’s?	695	79.1
3	2	What treatments are helpful for reducing balance problems and falls in people with Parkinson’s?	690	78.5
4	21	What helps improve the dexterity (fine motor skills or coordination of small muscle movements) of people with Parkinson’s so they can do up buttons, use computers, phones, remote controls etc?	673	76.6
5	4	What treatments would ensure the medications were equally effective each day (prevented/managed wearing off, variability, on/off states) in people with Parkinson’s?	667	75.9
6	7	What best treats mild cognitive problems such as memory loss, lack of concentration, indecision and slowed thinking in people with Parkinson’s?	662	75.3
7	1	What treatments are helpful in reducing tremor in people with Parkinson’s?	645	73.4
8	23	What is the best treatment for stiffness (rigidity) in people with Parkinson’s?	626	71.2
9	3	Is it possible to identify different types of Parkinson’s, for example, tremor dominant? And can we tailor treatments best according to these different types?	624	71.0
10	6	What is helpful for improving the quality of sleep in people with Parkinson’s?	615	70.0

* Number 1 in the majority of subanalyses therefore, listed above Q9.

**Table 3:** The summary statistics of all priority questions from pooled analysis of all sites and participants

Descriptive statistics for all questions							
(n=879)							
Rank	Question no	Question	Median	IQR1	IQR2	N who ranked ≥7	% Agreement
7	1	What treatments are helpful in reducing tremor in people with Parkinson’s?	8	6	9	645	73.4
3	2	What treatments are helpful for reducing balance problems and falls in people with Parkinson’s?	8	7	9	690	78.5
9	3	Is it possible to identify different types of Parkinson’s, for example, tremor dominant? And can we tailor treatments best according to these different types?	8	6	9	624	71.0
5	4	What treatments would ensure the medications were equally effective each day (prevented/managed wearing off, variability, on/off states) in people with Parkinson’s?	8	7	9	667	75.9
24	5	Would the monitoring of dopamine levels in the body (eg, with blood tests) be helpful in determining medication timing and amount (dose)?	7	6	8	532	60.5
10	6	What is helpful for improving the quality of sleep in people with Parkinson’s?	7	6	9	615	70.0
6	7	What best treats mild cognitive problems such as memory loss, lack of concentration, indecision and slowed thinking in people with Parkinson’s?	8	7	9	662	75.3
13	8	What treatments are helpful in reducing urinary problems (urgency, irritable bladder, incontinence) in people with Parkinson’s?	7	6	9	580	66.0
1	9	What drug treatments are best for the different stages of Parkinson’s?	8	7	9	695	79.1
19	10	What approaches are helpful for reducing stress and anxiety in people with Parkinson’s?	7	6	8	557	63.4
12	11	What treatments are helpful for reducing dyskinesias (involuntary movements, which are a side effect of some medications) in people with Parkinson’s?	7	6	8	589	67.0
11	12	What best treats dementia in people with Parkinson’s?	8	6	9	607	69.1
23	13	What interventions are effective for reducing or managing unexplained fatigue in people with Parkinson’s?	7	6	8	539	61.3
13	14	What best helps prevent or reduce freezing (of gait and in general) in people with Parkinson’s?	7	6	9	580	66.0
16	15	What treatments are helpful for swallowing problems (dysphagia) in people with Parkinson’s?	7	6	9	566	64.4
19	16	What is the best method of monitoring a person with Parkinson’s response to treatments?	7	6	8	557	63.4
26	17	What training, techniques or aids are needed for hospital staff, to make sure patients with Parkinson’s get their medications correctly and on time?	7	5	8	525	59.7
18	18	What treatments are helpful in reducing bowel problems (constipation, incontinence) in people with Parkinson’s?	7	6	8	562	63.9
1	19	What is the best type and dose of exercise (physiotherapy) for improving muscle strength, flexibility, fitness, balance and function in people with Parkinson’s?	8	7	9	695	79.1
15	20	Can medications be developed to allow fewer doses per day for people with Parkinson’s? (For example combinations of medications in one pill, slow release pills)	7	6	9	577	65.6
4	21	What helps improve the dexterity (fine motor skills or coordination of small muscle movements) of people with Parkinson’s so they can do up buttons, use computers, phones, remote controls etc?	8	7	9	673	76.6
27	22	What treatments are effective in reducing hallucinations (including vivid dreams) in people with Parkinson’s?	7	5	8	468	53.2
8	23	What is the best treatment for stiffness (rigidity) in people with Parkinson’s?	8	6	9	626	71.2
21	24	At which stage of Parkinson’s is deep brain stimulation (a surgical treatment that involves implanting a ‘brain pacemaker’ that sends signals to specific parts of the brain) most helpful?	7	6	8	553	62.9
25	25	What training to improve knowledge and skills do informal carers (family and friends) need in order to best care for people with Parkinson’s?	7	6	8	528	60.1
16	26	What is the best treatment for pain in people with Parkinson’s?	7	6	8	566	64.4
22	27	What speech therapy techniques are helpful for communication problems in people with Parkinson’s?	7	5	8	542	61.7

**Table 4:** Top 10 added questions from thematic analysis

Question no	Added question	Count (%)
28	What is the best treatment for low-mood/depression in PwP?	21 (14.5)
29	Which non-medication alternative therapies (eg, hypnosis, acupuncture etc) help to treat or manage Parkinson’s?	13 (9)
30	How can Parkinson’s be identified earlier?	12 (8.3)
31	Which genes predict Parkinson’s, the severity of progression, and the likelihood of developing non-motor complications?	9 (6.2)
32	What is the best way to educate PwP on living with Parkinson’s, managing symptoms, and accessing high quality information/support?	8 (5.5)
33	How can healthcare professionals be better educated in caring for, treating and monitoring PwP to ensure high quality care is always given (in both primary and secondary settings)?	8 (5.5)
34	Which dietary supplements in particular cannabis, help manage Parkinson’s?	7 (4.8)
35	What effect do medications for other conditions (eg, beta-blockers, chemotherapy), have on Parkinson’s symptoms and how can this be monitored more effectively?	6 (4.1)
36	What role does gut health (including use of probiotics) have in Parkinson’s?	6 (4.1)
37	How can apathy be more easily identified and managed in Parkinson’s?	6 (4.1)

PwP, people with Parkinson’s.

**Table 5:** Ranking results of priority questions from all subgroup analyses

Question no	Final top 10 s		Participant type		Disease duration				Education level				Living arrangements			Geographical location			Gender		Economic status	
	CENTRE-PD (n=879)	Deane *et al*^19^ (focus group n=27)	HCP (n=112)	PwP (n=511)	0–3 years (n=146)	3–5 years (n=115)	5–6 years (n=126)	9+ years (n=103)	Level 1–2 (n=64)	Level 3–4 (n=135)	Level 5–6 (n=128)	Level 7–8 (n=155)	Living Independently (n=331)	Supported by Family (n=123)	Supported by Carers (n=25)	UL (n=303)	EKUT (n=176)	UOXF (n=400)	Male (n=312)	Female (n=192)	Above Poverty Line (n=372)	Below Poverty Line (n=73)
1	seventh		second	eighth	third					tenth	seventh	seventh	sixth	seventh		third	tenth	tenth	fourth		tenth	fourth
2	third	first	first	third	seventh	fourth	first	fourth	sixth	third	fifth	fifth	sixth	third	second	first	eighth	fourth	third	sixth	fifth	second
3	ninth	fourth	eighth	ninth		seventh	fourth				third	ninth	fourth					fifth	eighth	ninth	sixth	
4	fifth		fifth	fourth	sixth	sixth	sixth	second	tenth	fifth	fourth	third	fifth	fourth	eighth	fourth	second	eighth	fifth	fifth	third	tenth
5															fourth							
6	tenth	eighth		tenth		eighth	ninth	eighth			eighth	tenth		second				sixth		seventh	eighth	
7	sixth	sixth	third	sixth	fourth	tenth	eighth	tenth		eighth	sixth	eighth	eighth	ninth	third	fifth	thirrd	seventh	sixth	eighth	sixth	sixth
8		tenth								ninth												
9	first		ninth	second	first	second	third	third	second	fourth	second	first	second	fifth	fifth	second	fourth	first	second	second	second	third
10		second							eighth													
11		third			tenth			seventh	fifth													
12		fifth																				
13											tenth						ninth					
14			sixth													tenth						
15			seventh												ninth	ninth						
16		seventh					tenth		seventh					sixth	seventh			ninth				ninth
17																						
18																						
19	first			first	second	first	first	first	first	first	first	second	first	first	first	sixth	first	first	first	first	first	first
20					eighth	fifth		fifth	ninth	fifth			tenth				seventh		tenth	tenth		eighth
21	fourth	ninth	fourth	fifth	fifth	third	fifth	ninth	third	second	ninth	fourth	third	tenth	tenth	seventh	fifth	third	seventh	third	fourth	sixth
22																						
23	eighth		ninth	seventh	ninth	ninth	seventh	sixth	fourth	seventh		sixth	ninth	eighth	sixth	seventh	fifth		ninth	fourth	ninth	fifth
24																						
25																						
26																						
27																						

Colour Key: Dark Blue - ranked first; Blue - ranked second; Light Blue - ranked third.

EKUT, University of Tübingen; HCP, healthcare professional; PwP, people with Parkinson’s; UL, University of Luxembourg; UOXF, University of Oxford.

### Alignment of PwP and HCP priorities

Of the included participants, there were 511 PwP and 112 HCPs; they shared eight top 10 research priorities (online supplemental material table 23). The number one priority for HCP was Q2 (‘hat treatments are helpful for reducing balance problems and falls in people with Parkinson’s?’) (88% agreement), which was ranked third by PwP. For PwP, the number one priority was Q19 (79% agreement). Q6 (‘What is helpful for improving the quality of sleep in people with Parkinson’s?’; 68% agreement) ranked 10th; neither made the HCP top 10 (joint 13th). Priorities ranked in the top 10 by HCP but not by PwP were Q14 (‘What best helps prevent or reduce freezing (of gait and in general) in people with Parkinson’s?’) (18th in PwP) and Q15 (‘What treatments are helpful for swallowing problems (dysphagia) in people with Parkinson’s?’) (23rd in PwP). The K score between HCP and PwP on the final top 10 was 0.682, or 68.2%, representing a substantial strength of agreement (p<0.001). In MWW tests to compare distributions of priority questions, PwP had a statistically significant lower mean rank, or lower distribution, in several questions compared with HCP (p=0.00–0.041).

### Disease duration

In the 490 responses from PwP, median disease duration was 5 years (IQR 3–9) which was similar across sites (medians: UL 5, EKUT, 6, UOXF 5), with a range of 0–41 years. To compare the effect disease duration had on the scoring of priority questions, disease duration was divided into quartiles: 0–3 years (n=146), 3–5 years (n=115), 5–9 years (n=126) and 9+ years (n=103). All quartile groups shared seven of the top 10 questions.

Between 0–3 years and 5–9 years, there was moderate agreement (k=52.4%, p=0.007), both of which had unique selections: 0–3 years was the only group to select Q1 (‘What treatments are helpful in reducing tremor in people with Parkinson’s?’) and omit Q6; 5–9 years were the only to select Q16 (‘What is the best method of monitoring a person with Parkinson’s response to treatments?’) and not include Q20 (‘Can medications be developed to allow fewer doses per day for people with Parkinson’s?’). There was good interrater reliability in other group analyses (K=68.2%–84.1% p<0.001). KW testing did not find any statistically significant differences in distribution of scores between these quartile groups (online supplemental material table S24).

### Education level

European guidance on Education Levels was used^30^ and 482 PwP gave education information: group 1 (level 1–2, n=64); group 2 (level 3–4, n=135); group 3 (level 5–6, n=128); group 4 (level 7–8, n=155). Only five questions were consistently in the top 10 for all groups. Q19 was top priority for groups 1–3, but was second for group 4, after Q9. Rater reliability between the two highest education groups was excellent (K 84.1%, p=0.00) although order and distribution differed. The reliability between group 1 and other groups was less certain (20.6%–52.4%, p=0.007–0.285). In KW testing, group 1 had a significant higher distribution for three questions compared with other groups and group 4 had a statistically significant lower distribution than other groups for multiple questions (online supplemental material tables S25–S43).

### Living arrangements

The majority of PwP were still living independently at home (n=331), followed by living at home supported by family members (n=123). Despite best efforts to increase responses from residential and nursing homes, response levels remained very low (n=6). They were grouped with participants living at home supported by carers (n=19) and analysed as supported by carer (n=25).

Q19 was top priority for all groups and between the subgroups, seven of the priorities were the same. There was substantial agreement (K=68.2%), between ‘Independent’ and ‘Supported by Family’ participants (p<0.001), and ‘Supported by Family’ and ‘Supported by Carer’ (p=0.007). Agreement between ‘Independent’ and ‘Supported by Carer’ was moderate, (52.4%, p<0.001). KW testing found significantly different distributions between ‘Supported by Carer’ and other groups for Q5, ‘Would the monitoring of dopamine levels in the body (eg, with blood tests) be helpful in determining medication timing and amount (dose)?’, (vs independent, p=0.002; vs supported by family, p=0.005) and Q17, ‘What training, techniques or aids are needed for hospital staff, to make sure patients with Parkinson’s get their medications correctly and on time?’ (vs independent, p=0.41). In both, there was a higher median, IQR and distribution of scoring from the carer group (online supplemental material tables S44–S47).

### Local institute/geographical location

The response by institute was; UL, n=303; EKUT, n=176 and UOXF, n=400. The number one priority for EKUT was Q19. UOXF had Q19 and Q9 as joint first. The number one priority at UL was Q2; Q19 was ranked sixth by UL. There was substantial agreement between UL and EKUT, sharing 8 of 10 priorities (K=68.2%, p<0.01). Between UOXF and UL, and UOXF and EKUT, agreement was moderate (K=52.4%, p=0.007 for both comparisons), sharing 7 out of 10 priorities as the only institute to include Q6 and omit Q23 ‘What is the best treatment for stiffness…?’. There were significant differences in the distribution of scores between the centres in KW testing. UL was significantly different in 10 questions, tending to have a higher scoring distribution (p=0–0.035). UOXF were significantly different in 10 questions, usually with a lower scoring distribution (p=0–0.048) (online supplemental material tables S48–S73).

### Gender

The PwP group was predominantly male (n=312) (female, n=192; Prefer not to say, n=7). Women and men with Parkinson’s shared 9 of the top 10 priorities: both rated Q19 as the number one priority. Men rated Q1 as fourth, but this did not feature in the women’s top 10 (14th). Women had Q6 at seventh however, this did not feature in the men’s top 10 (11th). There was excellent agreement in the final top 10 priorities between women and men with Parkinson’s (K=84.1% p<0.001). In MWW testing, comparing men to women, women had a higher mean rank in 12 of the priority questions (p=0.001–0.049).

### Economic status

The majority of PwP were living above the poverty line (n=372). People above and below poverty shared 8 of the top 10 priorities and Q19 was number one priority for both groups. There was significant reliability between groups (K=68.2% p<0.001). Above poverty line ranked Q6 and Q3 (‘Is it possible to identify different types of Parkinson’s, for example, tremor dominant? And can we tailor treatments best according to these different types?’) in the top 10; below poverty line ranked Q16 and Q20 instead. MWW found significant differences in the distribution of two questions, Q5 (p=0.04) and Q16 (p=0.03).

### Ethnicity

91.6% (n=217) of UOXF PwP responders were white ethnicity. Due to limited responses from Black, Asian and minority ethnic groups (BAME), responses were grouped together but only 11 participants made up the BAME group (excluding non-identifiable text responses from ‘Other’ group such as ‘Welsh’). It was not possible to be certain of the top 10 priorities for BAME group or individual ethnicity groups as the numbers were too small to compare nor draw any conclusions from.^31^

### Comparison with the 2014 Parkinson’s PSP

Comparing the CENTRE-PD top 10 to the final top 10 published by Deane *et al*^19^ showed poor agreement, with only 5 of the top 10 questions shared across both studies (K=20.6%, p=0.285). There was better agreement between the interim ranking round of the 2014 study and the CENTRE-PD top 10, sharing seven of the top 10 (K=52.4%, p=0.007).^19^

### Added questions

A total of 243 participants added a statement/question during the survey for thematic analysis. Like the 2014 group, questions pertaining to a cure were removed as this is the overarching theme of Parkinson’s research and would potentially take merit from other important questions. Other exclusion reasons included: the statement was an unclear question (eg, ‘hair loss’) or it was an information request (eg, ‘is deep brain stimulation available on the National Health Service?’) (figure 1). A total of 145 entries remained. The most common recurring theme from the survey was related to depression/low mood in Parkinson’s (table 4).

### Potential for selection bias

Strong evidence shows that local institution related to exclusion from the study sample (p<0.001) where one patient from Tubingen was excluded. Inclusions rates between PwP and HCP was similar (p=0.17). Within PwP, there was little evidence that age (p=0.08), gender (p=0.64), disease duration (p=0.76), education level (p=0.016), living arrangements (p=0.64) and economic status (p=0.64) related to exclusion from the study sample. This suggests potential for selection bias with regard to local institution but not much evidence for other patient characteristics.

## Discussion

Our study identified Q19, ‘What is the best type and dose of exercise (physiotherapy) for improving muscle strength, flexibility, fitness, balance and function in people with Parkinson’s?’, as the most important question in the majority of analyses, as well as coming joint first in the main pooled analysis. Therefore, it was deemed the most important research question. This is the first PSP to consider the effect socioeconomic factors may have on the prioritisation of research questions. There was no substantial evidence that socioeconomic factors affected the top priority but there was an observable difference between the prioritisation of other questions in the top 10. The groups based on disease duration, educational attainment and living arrangements had the least agreement in the top 10 priorities (K<60%). There were observable differences in how HCP and PwP ranked the research priorities.

### HCP versus PwP

HCP rated questions on freezing of gait and swallowing problems as more important in contrast with PwP who considered physiotherapy and improving sleep more important. Gait freeze and dysphagia symptoms are likely to have significant effects on patient’s safety which could explain why HCP felt these should be included. These symptoms are more likely to be experienced later on in disease progression and not all PwP will experience them. The PwP taking part in this study were of modest disease duration (median 5 years) and thus unlikely to have reached a point in their disease where these symptoms are more common. Contrastingly, physiotherapy is currently the best treatment option for PwP for longevity of independence.^32^ Sleep improvement could not only improve movement symptoms but reduce the fatigue experienced by an estimated 50% of PwP.^33^

Interestingly, the only analysis in which Q19 did not appear in the top 10 at all, was the HCP survey responses, suggesting HCP are having an impact on the prioritisation of research which does not necessarily correlate with what patients and the public prioritise. This could be due to factors such as their knowledge of disease manifestations and treatment options, funding requirements, career paths, current research projects or personal interests. However, this misalignment could lead to less research being conducted into topics important to patients, highlighting the importance of patient and PPI in research at all stages of research methodology. It could also indicate the need to ensure that in research projects and policy development, a broader range of stakeholders should be included to ensure the preferences of HCP do not significantly overshadow that of patients. When making comparisons, it should be considered that responses between HCP and PwP will have factors influencing their responses (eg, disease duration).

### Disease duration

The disease duration quartile groups shared seven of the top 10 priorities. Reducing balance problems and falls was ranked ahead of physiotherapy by those with a disease duration of 5–9 years, this potentially correlates with the time period in which people typically begin to experience these symptoms.^34^ This was the only group to select monitoring response to treatments, potentially correlating with the changes in medication efficacy and increasing complications as the disease progresses. Levodopa provides symptom management for most PwP; however, long-term use can have complications such as dyskinesias, impulse control disorders, motor fluctuations and reduced improvements over time.^35 36^ Drug treatments for the different stages of Parkinson’s was selected as the highest priority for those in 0–3 years disease duration category. Newly diagnosed PwP (0–3 years) may have rated this higher as they begin to notice the progression of the disease within themselves and the need to increase their Parkinson’s medication. They were also the only group to select reducing tremor as a priority and not include improving sleep.

### Educational level

The distribution of scores showed a statistically significant variance depending on the educational level of responder. Moreover, only 5 of the questions in the top 10 were shared by all education groups. Level 1–2 shared five of the top questions with level 5–6 and six questions with level 7–8. In contrast, level 5–6 shared 9 of the top 10 questions with level 7–8. The level 1–2 had three priorities which did not feature in the other groups: reducing stress and anxiety, treating dyskinesias and monitoring response to treatments. Depression and anxiety are higher in low educational levels which could explain this.^37^ People with a level 7–8 educational achievement did not have Q19 as their first priority, instead they selected drug treatments for different stages as their top priority potentially due to differences in quality of life and health literacy.^38^ The other disparities between the groups did not necessarily have a correlation to education. Arguably, results could be biased towards those of a higher education level who may have completed the questionnaire more thoroughly.

### Living arrangements

When grouped by living arrangement, PwP shared 7 of the top 10 priorities. Those living independently wanted to know about different types of Parkinson’s and the development of medications requiring fewer doses. Neither are directly related to symptom management which correlates with people living independently. The carer group prioritised monitoring dopamine levels and monitoring the response to treatments, possibly due to medication losing efficacy over time or the progression of Parkinson’s.^35 36^ The PwP needing carers or living in a care home were the only PwP to select the question pertaining to dysphagia, correlating with the previous point that these are likely experienced at later disease progression. However, despite pooling PwP supported by carers and those living in care or nursing homes, there was a small sample size (n=25); the majority of PwP were independent (n=321).

### Geography, gender and poverty status

UOXF shared 7 of the top 10 priorities with the other centres and shared Q19 as the top priority with EKUT. UL selected Q2 as their top priority; UL had the highest response from HCP (24% of participants) which could explain why the top priority for this centre was more aligned with that of HCP than the PwP analyses (EKUT 3% of responses were HCP and UOXF had 8% HCP). There was closer agreement between EKUT and UL, sharing eight of the top 10 priorities. Many UL responders were living in Germany which may explain the slightly higher rater reliability between these centres. UOXF has been working on sleep projects which has likely raised awareness within the cohort, as UOXF was the only centre to select Q6.

Nine priorities were shared across groups in both research participation and gender, and eight were shared across economic status groups: with no category changing the number one priority. Ethnicity was difficult to expand on due to 91.6% (n=217) of UOXF PwP responders being white ethnicity. The latest UK census figures^39^ indicate that 87% of UK residents are white so this is not reflective of the UK population.

### Comparison with the 2014 Parkinson’s PSP

When comparing the CENTRE-PD top 10 (n=879, 58% PwP) to the previous publication from 2014, the interim ranking round (n=475, 72% PwP) was more consistent with the updated priorities than the nominal group (n=27, 34% PwP). This could be because the group had more HCP and other professionals influencing the final decision. Q19 was unique to the CENTRE-PD top 10, indicating a change in priorities since 2014,^19^ potentially due to recent promotions on exercise in Parkinson’s. Interestingly, comparing our added questions with the original 94 question survey showed some overlap (specifically about depression, genes and apathy), questioning accuracy of the top 10 priorities list.^19^

Beneficially, this was an international study, but a namely limitation is that the majority of our responders with Parkinson’s were independent, white European, economically comfortable and highly educated, which does not make it a truly representative sample of the populations included. Furthermore, the 27 core questions were generated from a UK sample, querying the results validity. For future PSP groups, it might be advisable to enrol care homes, general practitioners and charities as stakeholders (with large participant databases) to encourage participant recruitment. This could enable further comparisons to be made between groups, for example, comparing responses between disability severity, and to reduce response variability between stakeholder groups (table 1). Future groups should consider the translation to more languages and distribution of the survey to a greater international audience, thanks to the ease and affordability of web-based instruments. These limitations were similar to the previous Parkinson’s PSP, despite efforts from both studies to recruit from all groups of people.

## Conclusions

Overall, this study has given a useful steer as to how socioeconomic factors may influence the priorities and decision making during PSP There was a general consensus on the top 10 across the various socioeconomic groups with the majority of groups agreeing that physiotherapy was the most important priority for PwP. This gives another avenue for future research, showing that physical activity is of very high importance to PwP, and not only does this have other health benefits, but it may be more deliverable and cheaper (compared with drug trials).

Of the socioeconomic comparisons, education level, disease duration and living arrangements, had the most impact on the prioritisation of questions. Other socioeconomic factors including poverty and gender did not significantly affect the final prioritisation.

HCP did not identify the most important priority to PwP, so care should be taken with sampling (ie, through stratification) to ensure future PSP or clinical decision groups do not miss key priorities for those living with Parkinson’s, in deference to the priorities of professionals which may not always align with patient views.

## Supplementary material

10.1136/bmjopen-2021-049530 online supplemental file 1

## Data Availability

Data are available in a public, open access repository. Data are available on reasonable request. All data relevant to the study are included in the article or uploaded as online supplemental information.
