# Supplementary material for: Exploration of whether socioeconomic factors affect the results of priority setting partnerships: updating the top 10 research priorities for the management of Parkinson’s in an international setting
Source: BMJ Open. 2022 Jun 29;12(6):e049530. doi: 10.1136/bmjopen-2021-049530 (PMC9251108; doi:10.1136/bmjopen-2021-049530)
Supplement: online supplemental file 1 [file bmjopen-2021-049530supp001.pdf]

CENTRE-PD Top 10 Supplementary Material

Table of Contents

CENTRE-PD Top 10 Supplementary Material **1**

Table 1. Glossary 3
Figure 1. Top 10 from 2014 Final Prioritisation 4
Figure 2. Top 26 from 2014 Interim Prioritisation 5
Table 2. PSP Group Sizes 6
Table 3. Survey Responses by Participant Type and Local Institute 7
Table 4. Healthcare professional responder's by role type 7
Figure 3. Histogram and Statistics for Duration of Disease 8
Table 5. Ethnicity Count for UOXF 8
Figure 4. Bar Chart of PwP Education Level Frequencies 8
Figure 5. Bar Chart of PwP Living Arrangements 9

Box Plot and Whiskers for Analyses 9

Figure 6. Box Plot and Whisker for Pooled Survey Round 9
Figure 7. Box Plot and Whisker for Priority Questions by HCP and PwP 10
Figure 8. Box Plot and Whisker by Disease Duration 10
Figure 9. Box Plot and Whisker by Education Level 11
Figure 10. Box Plot and Whisker by Living Arrangements 11
Figure 11. Box Plot and Whisker of Survey Results by Local Institute 12
Figure 12. Box Plot and Whisker Comparing Results by Gender 12
Figure 13. Box Plot and Whisker by Economic Status 13
Figure 14. Box Plot and Whisker Comparing Results by Grouped Ethnicity 13

Top 10 by Sub group 14

Table 6. Top 10 by PwP and HCP. 14
Table 7. Top 10 by Disease Duration Quartiles 15
Table 8. Top 10 by Education Level 16
Table 9. Top 10 by Living Arrangements 17
Table 10. Top 10 by Local Institute 18
Table 11. Top 10 Priorities by Gender 19
Table 12. Top 10 by Economic Status 20
Table 13. Top 10 by Ethnicity Groups 20

Kappa Tables 21

Table 14. Kappa Agreement between Disease Duration Groups 21
Table 15. Kappa Agreement between Education Levels 21
Table 16. Kappa test of Agreement by Living Arrangement (grouped) 21

Table 17. Kappa Test for Agreement between Local Institutes 21
Table 18. Kappa for Gender 22
Table 19. Kappa for Economic Status 22
Table 20. Kappa test between HCP and PwP 22
Table 21. Kappa test with Deane et al Top 10 by Focus group 22
Table 22. Kappa test with Deane et al by interim ranking 23

Distribution Analyses 24

Table 23. Distribution Comparisons of each question by subgroup 24
Table 24. Pairwise comparison of significant KW Disease Duration for Question 4. 28
Table 25. Pairwise comparison of significant KW Education Level for Question 1. 28
Table 26. Pairwise comparison of significant KW Education Level for Question 2. 28
Table 27. Pairwise comparison of significant KW Education Level for Question 6. 28
Table 28. Pairwise comparison of significant KW Education Level for Question 8. 29
Table 29. Pairwise comparison of significant KW Education Level for Question 10. 29
Table 30. Pairwise comparison of significant KW Education Level for Question 11. 29
Table 31. Pairwise comparison of significant KW Education Level for Question 12. 29
Table 32. Pairwise comparison of significant KW Education Level for Question 15. 30
Table 33. Pairwise comparison of significant KW Education Level for Question 16. 30
Table 34. Pairwise comparison of significant KW Education Level for Question 17. 30
Table 35. Pairwise comparison of significant KW Education Level for Question 18. 30
Table 36. Pairwise comparison of significant KW Education Level for Question 19. 31
Table 37. Pairwise comparison of significant KW Education Level for Question 20. 31
Table 38. Pairwise comparison of significant KW Education Level for Question 21. 31
Table 39. Pairwise comparison of significant KW Education Level for Question 22. 31
Table 40. Pairwise comparison of significant KW Education Level for Question 23. 32
Table 41. Pairwise comparison of significant KW Education Level for Question 25. 32
Table 42. Pairwise comparison of significant KW Education Level for Question 26. 32
Table 43. Pairwise comparison of significant KW Education Level for Question 27. 32
Table 44. Pairwise comparison of significant KW Living Arrangements for Question 5. 33
Table 45. Pairwise comparison of significant KW Living Arrangements for Question 7. 33
Table 46. Pairwise comparison of significant KW Living Arrangements for Question 15. 33
Table 47. Pairwise comparison of significant KW Living Arrangements for Question 17. 33
Table 48. Pairwise comparison of significant KW Local Institute for Question 1. 34
Table 49. Pairwise comparison of significant KW Local Institute for Question 2. 34
Table 50. Pairwise comparison of significant KW Local Institute for Question 4. 34
Table 51. Pairwise comparison of significant KW Local Institute for Question 5. 34
Table 52. Pairwise comparison of significant KW Local Institute for Question 6. 34
Table 53. Pairwise comparison of significant KW Local Institute for Question 7. 35
Table 54. Pairwise comparison of significant KW Local Institute for Question 8 35
Table 55. Pairwise comparison of significant KW Local Institute for Question 9 35

Table 56. Pairwise comparison of significant KW Local Institute for Question 10 ... 35
Table 57. Pairwise comparison of significant KW Local Institute for Question 11. ... 35
Table 58. Pairwise comparison of significant KW Local Institute for Question 12. ... 36
Table 59. Pairwise comparison of significant KW Local Institute for Question 13. ... 36
Table 60. Pairwise comparison of significant KW Local Institute for Question 14. ... 36
Table 61. Pairwise comparison of significant KW Local Institute for Question 15. ... 36
Table 62. Pairwise comparison of significant KW Local Institute for Question 16. ... 36
Table 63. Pairwise comparison of significant KW Local Institute for Question 17. ... 37
Table 64. Pairwise comparison of significant KW Local Institute for Question 18. ... 37
Table 65. Pairwise comparison of significant KW Local Institute for Question 19. ... 37
Table 66. Pairwise comparison of significant KW Local Institute for Question 20. ... 37
Table 67. Pairwise comparison of significant KW Local Institute for Question 21. ... 37
Table 68. Pairwise comparison of significant KW Local Institute for Question 22. ... 38
Table 69. Pairwise comparison of significant KW Local Institute for Question 23. ... 38
Table 70. Pairwise comparison of significant KW Local Institute for Question 24. ... 38
Table 71. Pairwise comparison of significant KW Local Institute for Question 25. ... 38
Table 72. Pairwise comparison of significant KW Local Institute for Question 26. ... 38
Table 73. Pairwise comparison of significant KW Local Institute for Question 27. ... 39

Table 1. Glossary

≥7	High Importance (greater or equal to 7 in rating).
BAME	Black, Asian and Minority Ethnicity
CUREC	Central University Research Ethics Committee
EKUT	Eberhard Karls University of Tübingen
HCP	Healthcare Professionals/Researchers in Health
IQR	Interquartile Range
JLA	James Lind Alliance
K	Kappa
KW	Kruskal-Wallis
MCI	Mild Cognitive Impairment
MWW	Mann-Whitney Wilcoxon
NGT	Nominal Group Technique
NICE	National Institute of Clinical Excellence
OPDC	Oxford Parkinson's Disease Centre
PPI	Patient Public Involvement
PSP	Patient Setting Priority
PwP	People with Parkinson's
REC	Research Ethics Committee
SBC	Supported by carers/in care home
SD	Standard Deviation
UL	University of Luxembourg
UOXF	University of Oxford
WHO	World Health Organisation

Figure 1. Top 10 from 2014 Final Prioritisation.

Table 3 Final prioritised and ranked uncertainties for the management of Parkinson's disease

**Overarching research aspiration: an effective cure for Parkinson's disease**	
1	What treatments are helpful for reducing balance problems and falls in people with Parkinson's?
2	What approaches are helpful for reducing stress and anxiety in people with Parkinson's?
3	What treatments are helpful for reducing dyskinesias (involuntary movements, which are a side effect of some medications) in people with Parkinson's?
4	Is it possible to identify different types of Parkinson's, eg, tremor dominant? And can we develop treatments to address these different types?
5	What best treats dementia in people with Parkinson's?
6	What best treats mild cognitive problems such as memory loss, lack of concentration, indecision and slowed thinking in people with Parkinson's?
7	What is the best method of monitoring a person with Parkinson's response to treatments?
8	What is helpful for improving the quality of sleep in people with Parkinson's?
9	What helps improve the dexterity (fine motor skills or coordination of small muscle movements) of people with Parkinson's so they can do up buttons, use computers, phones, remote controls etc?
10	What treatments are helpful in reducing urinary problems (urgency, irritable bladder, incontinence) in people with Parkinson's?

Figure 2. Top 26 from 2014 Interim Prioritisation

Uncertainty	PwP Score	Carer Score	F&F Score	HSCP Score	Total	Interim rank
What treatments are helpful in reducing tremor in people with Parkinson's?	93	83	92	91	359	1
What treatments are helpful for reducing balance problems and falls in people with Parkinson's?	92	93	80	94	359	1
Is it possible to identify different types of Parkinson's, eg, tremor dominant? And can we tailor treatments best according to these different types?	88	88	89	88	353	3
What treatments would ensure the medications were equally effective each day (prevented/managed wearing off, variability, on/off states) in people with Parkinson's?	89	94	88	81	352	4
Would the monitoring of dopamine levels in the body (eg, with blood tests) be helpful in determining medication timing and amount (dose)?	91	89	86	86	352	4
What is helpful for improving the quality of sleep in people with Parkinson's?	94	79.5	93	84	350.5	6
What best treats mild cognitive problems such as memory loss, lack of concentration, indecision and slowed thinking in people with Parkinson's?	87	91	77	89.5	344.5	7
What treatments are helpful in reducing urinary problems (urgency, irritable bladder, incontinence) in people with Parkinson's?	90	77	94	79	340	8
What drug treatments are best for the different stages of Parkinson's?	83	87	87	77.5	334.5	9
What approaches are helpful for reducing stress and anxiety in people with Parkinson's?	75	77	82	92	326	10
What treatments are helpful for reducing dyskinesias (involuntary movements, which are a side effect of some medications) in people with Parkinson's?	80	90	73.5	77.5	321	11
What best treats dementia in people with Parkinson's?	56	92	75	93	316	12
What interventions are effective for reducing or managing unexplained fatigue in people with Parkinson's?	78	65	85	85	313	13
What best helps prevent or reduce freezing (of gait and in general) in people with Parkinson's?	79	71.5	76	82	308.5	14
What treatments are helpful for swallowing problems (dysphagia) in people with Parkinson's?	66	74.5	81	80	301.5	15
What is the best method of monitoring a person with Parkinson's response to treatments?	81	52.5	83.5	83	300	16
What training, techniques or aids are needed for hospital staff, to make sure patients with Parkinson's get their medications correctly and on time?	53	86	64.5	89.5	293	17
What treatments are helpful in reducing bowel problems (constipation, incontinence) in people with Parkinson's?	77	85	90	40	292	18
What is the best type and doe of exercise (physiotherapy) for improving muscle strength, flexibility, fitness, balance and function in people with Parkinson's?	84	68	64.5	67.5	284	19
Can medications be developed to allow fewer doses per day for people with Parkinson's? (For example combinations of medications in one pill, slow release pills)	73	84	56	69	282	20
What helps improve dexterity (fine motor skills or coordination of small muscle movements) of people with Parkinson's so they can do up buttons, use computers, phones, remote controls etc?	85	59.5	73.5	54.5	272.5	21
What treatments are effective in reducing hallucinations (including vivid dreams) in people with Parkinson's?	52	79.5	71.5	61	264	22
What is the best treatment for stiffness (rigidity) in people with Parkinson's?	86	67	63	46	262	23
At which stage of Parkinson's is deep brian stimulation (a surgical treatment that involves implanting a 'brain pacemaker' that sends signals to specific parts of the brain) most helpful?	69	59.5	91	42	261.5	24
What training to improve knowledge and skills do informal carers (family and friends) need in order to best care for people with Parkinson's?	42	82	70	63.5	257.5	25
What is the best treatment for pain in people with Parkinson's?	82	54	60.5	57.5	254	26

F&F, family and friends; HSCP, health and social care professionals; PwP, people with Parkinson's.

Table 2. PSP Group Sizes

PSP Group	Year	Interim N=	Workshop N=	Note
Acne	2014	1573	43	
Eczema		493		
Vitiligo		461		
Alcohol-related Liver Disease	2017	230		
Anaesthesia and Perioperative Care	2015	1718	23	Mostly clinicians
Autism	2015	1266		
Bipolar	2016	2200	26	
Blood transfusion	2015	568	13	
Broken bones in old people	2018	209		
Carcinoma	2015	141	29	
Cellulitis	2017	353	28	
Childhood disability	2015	75	21	
Common conditions effecting hand and wrist	2017	261	21	
Contraception	2017	407	10	
Cystic Fibrosis	2017	677		
Dementia	2013	36	18	36 organisations
Depression	2016	1700	16	
Diabetes Mellitus Type 1	2011	47	23	
Diabetes Mellitus Type 2	2017	1500	26	
Hip and knee osteoarthritis	2013	266	25	
Digital Technology for Mental Health	2018	137	27	
Eczema	2012	514	40	
Emergency Medicine	2017	513	34	
Endometriosis	2017	1418	26	
Fibromyalgia	2017		18	
Head and Neck Cancer	2017	49	20	
Hidradenitis Suppurativa	2014	371	22	
Hypertension	2017	63	15	
Irritable Bowel Syndrome	2017		16	
Intensive Care Unit	2014	513		
kidney transplant	2016	256	20	
Idiopathic Intracranial Hypertension	2018	401	25	
Learning Difficulties	2017	361	25	
Lichen Sclerosis		954	29	
Life after stroke	2011	97	28	
Lyme Disease	2011	103	9	
Mesothelioma	2014	202	30	
Mild to Moderate Hearing Loss	2015	486	7	
Miscarriage	2017	2122	21	
Multiple Conditions in Later Life	2018		24	
Multiple Sclerosis	2013	669	35	
Neurodevelopmental Disorders	2017	177	31	
Neuro-oncology	2015	227	18	
Palliative and end of life care	2015	1331	24	
Parkinson's	2014	475	27	
Patient Safety in Primary Care	2017	447	22	
Pessary use for Prolapse	2017	278	23	
Physiotherapy	2018	635	27	
Pressure Ulcers	2013	141	27	
Preterm Birth	2014	537	34	
Prostate Cancer	2010		26	40 "groups" in first survey no interim data
Rare Inherited Anaemias	2018	120	31	
Scoliosis	2017	750	22	
Sight Loss and Vision	2015	664	12.9	Mean from 12 types of workshops, total 155 people
spinal cord injury	2016	293	20	
Stillbirth	2015	1118	18	Counted in photo from focus group
Stroke	2012	97	28	
Teenage and Young Adult Cancer	2018	174	25	
Tinnitus	2012	630	18	P
Urinary Incontinence	2008	11	13	11 "organisations"
Vitiligo	2010	230	47	6 observers
Womb Cancer	2016	253	23	
**Average (mean)**		**552**	**24**	
**Median**		**386**	**24**	
Data from 96 PSP groups which had published data on 24/OCT/2018				

Table 3. Survey Responses by Participant Type and Local Institute

		Local Institute			
		UL Count	EKUT Count	UOXF Count	Total
Participant Type	PwP	116	158	237	**511**
	Carer/Former Carer	1	1	36	**38**
	Friend/Family Member	113	10	73	**196**
	HCP	73	7	32	**112**
	Person with RBD	0	0	22	**22**
	**Total**	**303**	**176**	**400**	**879**

Table 4. Healthcare professional responder's by role type.

Health Care Professional Role	Count
Charity Worker	1
Dietician	1
Doctor	26
Educator	1
GP	3
Laboratory / Scientist	2
Neurologist	3
Neuropsychologist	1
Not Specified	8
Nurse or Research Nurse	17
Occupational Therapist	11
PhD Student	1
Physiotherapist	10
Psychiatrist	1
Psychologist	2
Research Administrator	1
Research Assistant	1
Researcher	4
Scientific Research Project Manager	3
Social Worker	2
Speech and Language Therapist	12
Student Nurse	1
**Total**	**112**

Figure 3. Histogram and Statistics for Duration of Disease

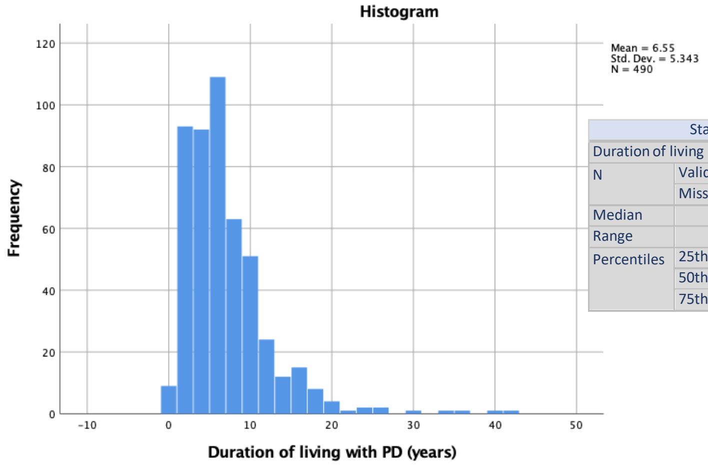

Statistics

Duration of living with PD (years)		
N	Valid	490
	Missing	21
Median		5
Range		41
Percentiles	25th	3
	50th	5
	75th	9

Table 5. Ethnicity Count for UOXF

Ethnic Group	Frequency	Percent (%)
Other/Please Specify	3	1.3
Asian/Asian British	6	2.5
Arab	1	0.4
Black/Black British	3	1.3
White	217	91.6
Mixed/multiple ethnic groups	2	0.8
Prefer not to say	5	2.1
**Total**	**237**	**100**

Figure 4. Bar Chart of PwP Education Level Frequencies

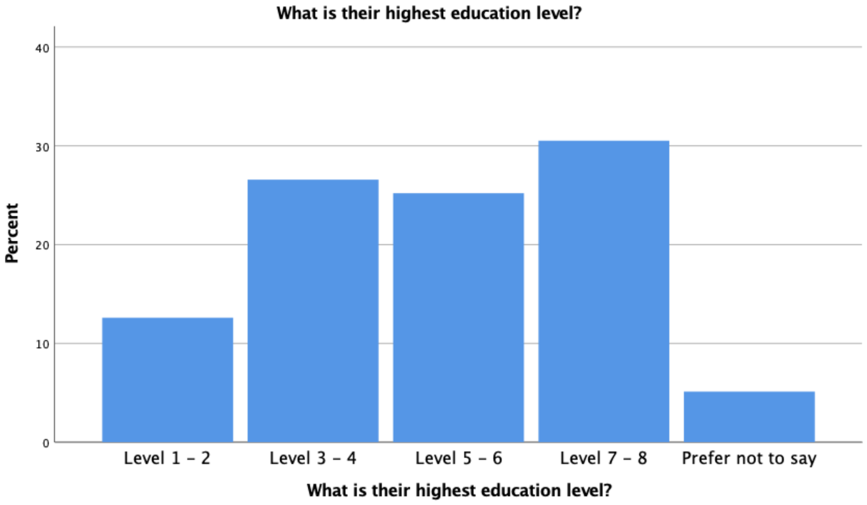

Figure 5. Bar Chart of PwP Living Arrangements

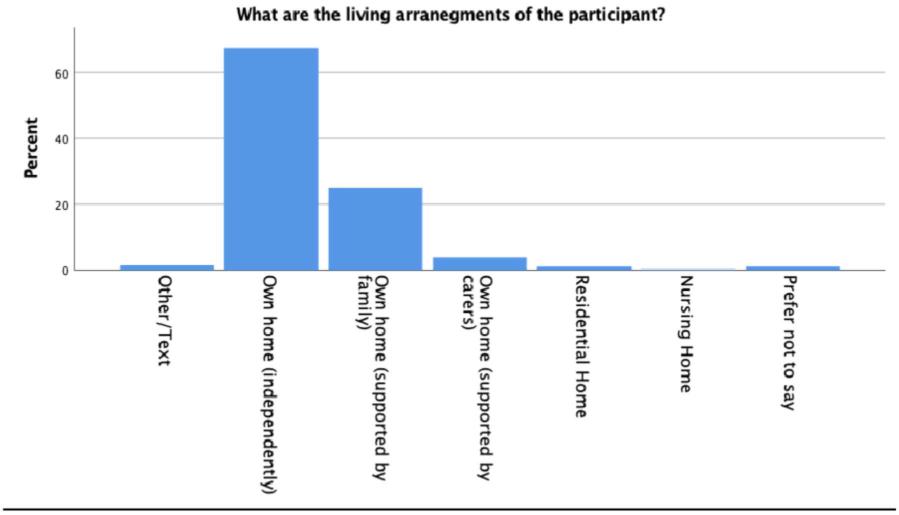

Box Plot and Whiskers for Analyses

Figure 6. Box Plot and Whisker for Pooled Survey Round

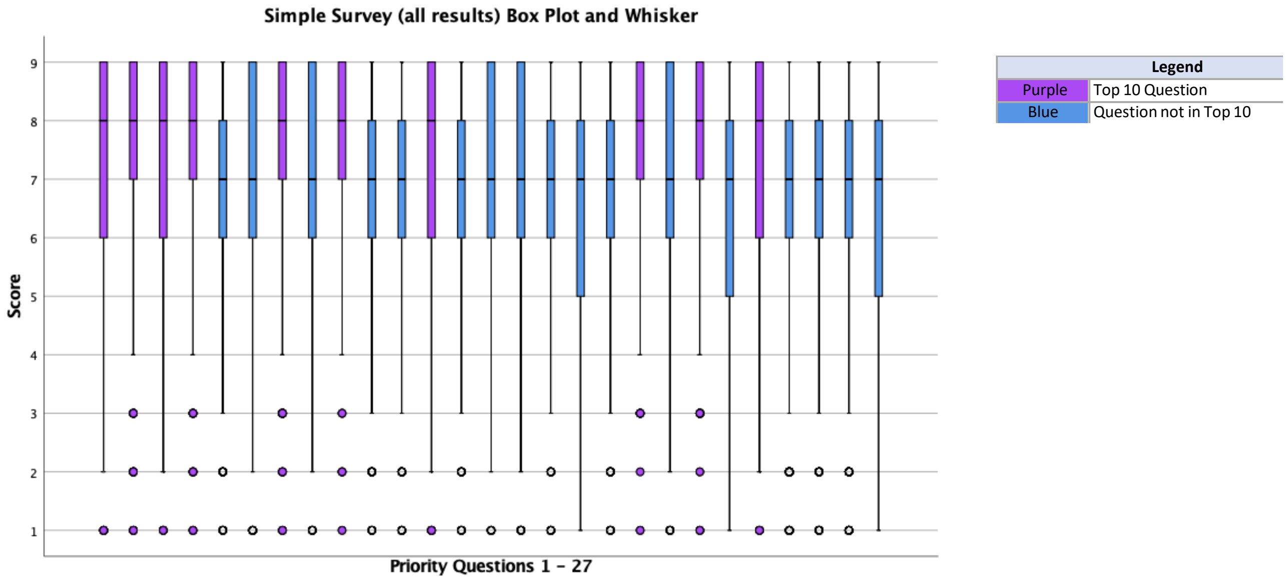

Figure 7. Box Plot and Whisker for Priority Questions by HCP and PwP

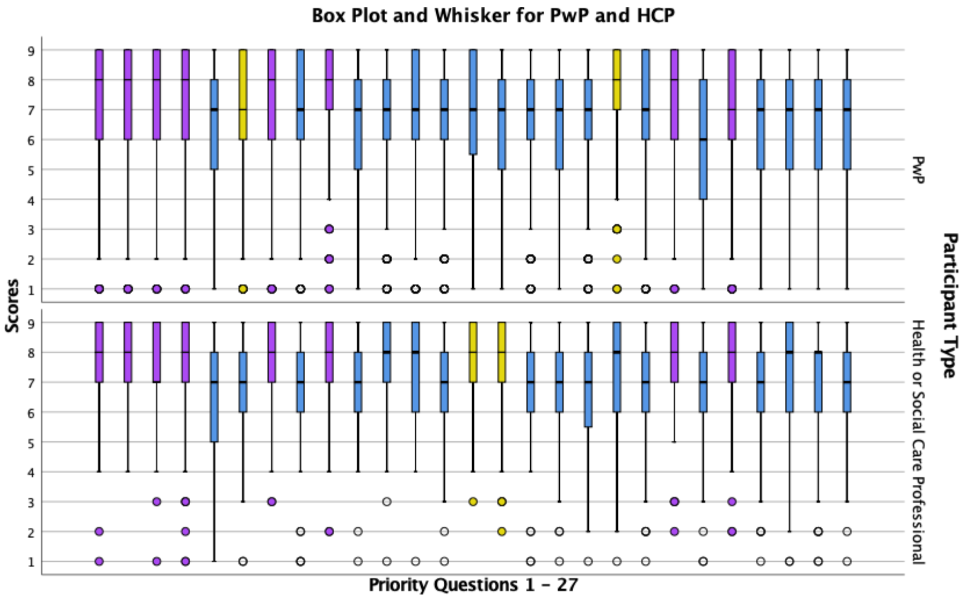

Legend	
Purple	Top 10 Question
Yellow	Disparity in Top 10 Between Groups
Blue	Question not in Top 10

Figure 8. Box Plot and Whisker by Disease Duration

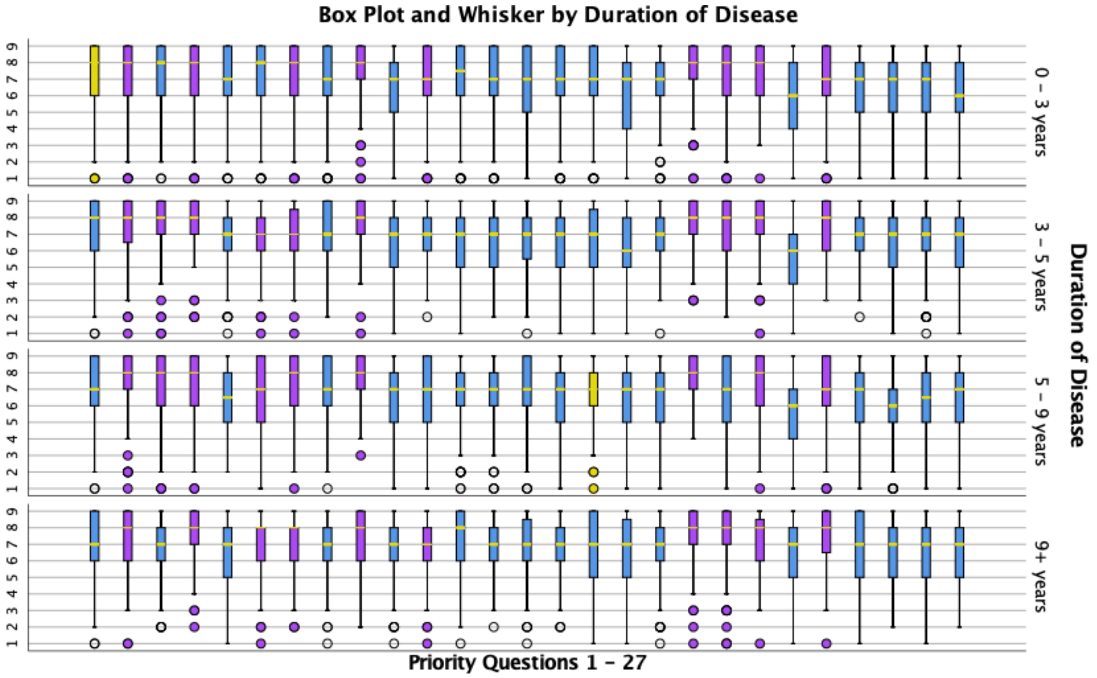

Legend	
Purple	Top 10 Question
Yellow	Disparity in Top 10 Between Groups
Blue	Question not in Top 10

Figure 9. Box Plot and Whisker by Education Level

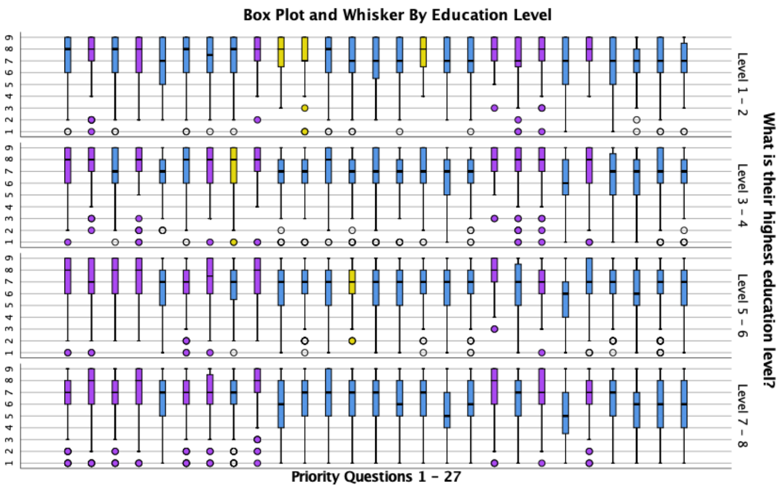

Legend	
Purple	Top 10 Question
Yellow	Disparity in Top 10 Between Groups
Blue	Question not in Top 10

Figure 10. Box Plot and Whisker by Living Arrangements

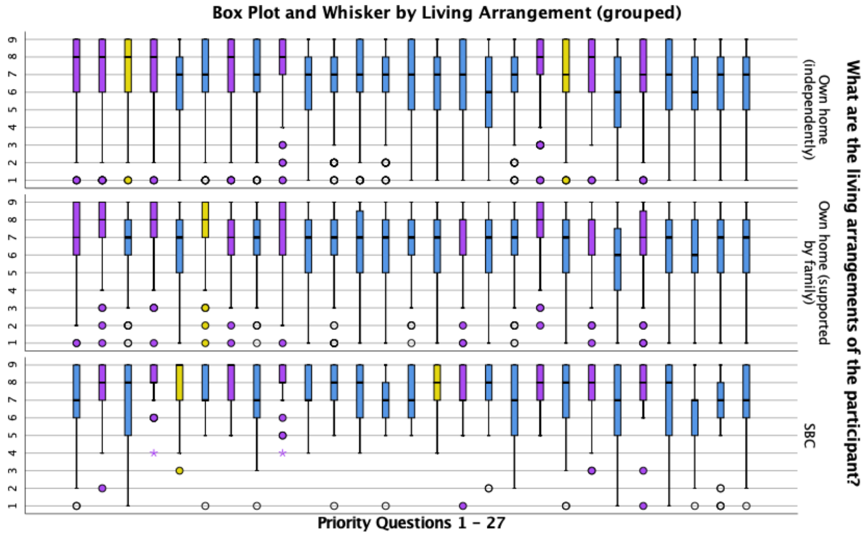

Legend	
Purple	Top 10 Question
Yellow	Disparity in Top 10 Between Groups
Blue	Question not in Top 10

Figure 11. Box Plot and Whisker of Survey Results by Local Institute

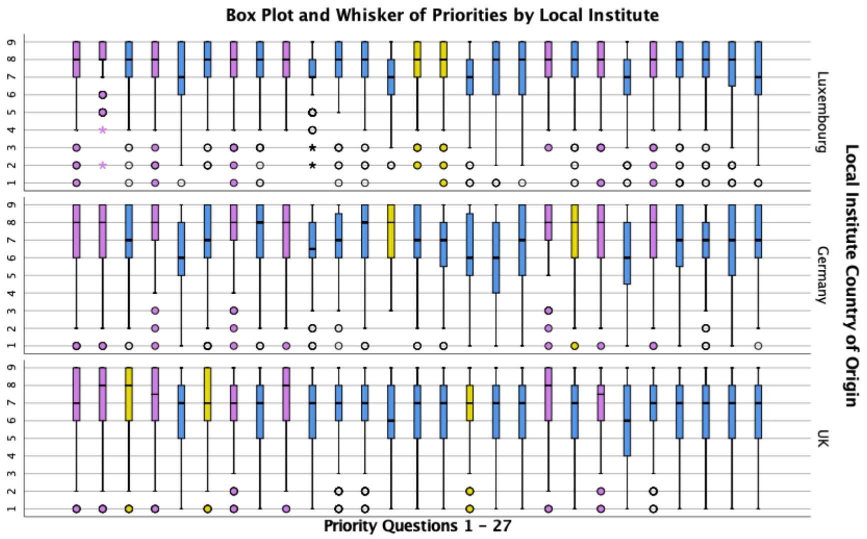

Legend	
Purple	Top 10 Question
Yellow	Disparity in Top 10 Between Groups
Blue	Question not in Top 10

Figure 12. Box Plot and Whisker Comparing Results by Gender

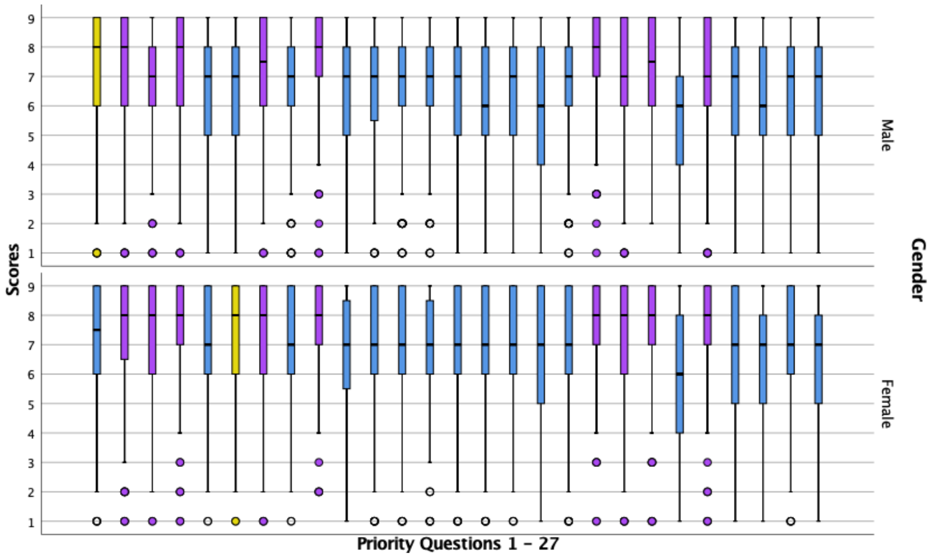

Legend	
Purple	Top 10 Question
Yellow	Disparity in Top 10 Between Groups
Blue	Question not in Top 10

Figure 13. Box Plot and Whisker by Economic Status

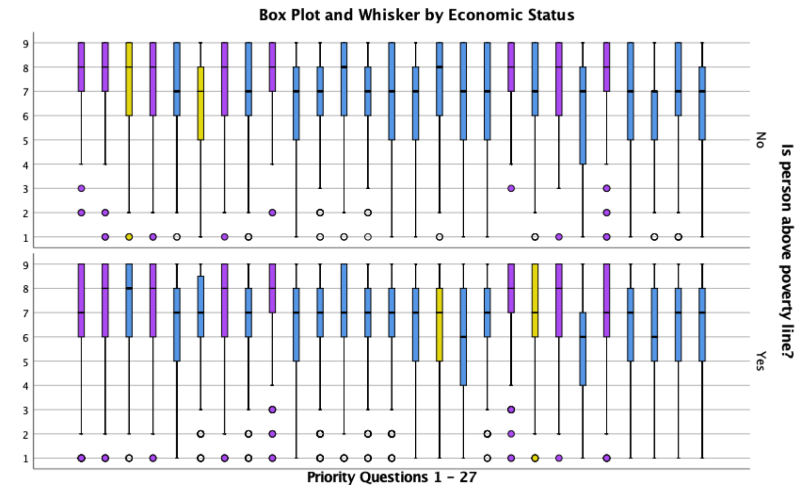

Legend	
Purple	Top 10 Question
Yellow	Disparity in Top 10 Between Groups
Blue	Question not in Top 10

Figure 14. Box Plot and Whisker Comparing Results by Grouped Ethnicity

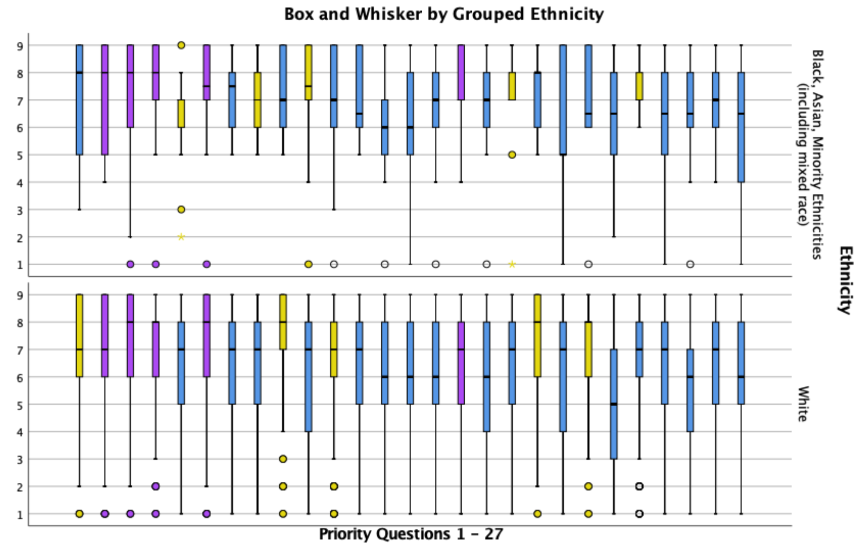

Legend	
Purple	Top 10 Question
Yellow	Disparity in Top 10 Between Groups
Blue	Question not in Top 10

Top 10 by Sub group

Table 6. Top 10 by PwP and HCP.

Descriptive Statistics PwP and HCP								
**HCP (n = 112)**								
		Range		Percentiles				
Rank	Question Number	Minimum	Maximum	25th	50th	75th	N who ranked ≥7	% Agreement
1	2	4	9	7	8.5	9	99	88
2	1	1	9	7	8	9	95	85
3	7	3	9	7	8	9	95	85
4	21	2	9	7	8	9	91	81
5	4	1	9	7	8	9	89	79
6	14	3	9	7	8	9	88	79
7	15	2	9	7	8	9	88	79
8	3	1	9	7	7	9	85	76
9	9	2	9	6.75	8	9	84	75
9	23	2	9	6.75	8	9	84	75
**PWP (n = 511)**								
		Range		Percentiles				
Rank	Question Number	Minimum	Maximum	25th	50th	75th	N who ranked ≥7	% Agreement
1	19	1	9	7	8	9	406	79
2	9	1	9	7	8	9	394	77
3	2	1	9	6	8	9	373	73
4	4	1	9	6	8	9	371	73
5	21	1	9	6	8	9	369	72
6	7	1	9	6	8	9	358	70
7	23	1	9	6	7	9	355	69
8	1	1	9	6	8	9	348	68
9	3	1	9	6	8	9	346	68
10	6	1	9	6	7	9	346	68

Table 7. Top 10 by Disease Duration Quartiles

0-3 years duration (n=146)

Rank	Question	Range: Minimum	Range: Maximum	Percentiles: 25th	Percentiles: 50th	Percentiles: 75th	N who ranked ≥7	% Agreement
1	9	1	9	7	8	9	118	80.8
2	19	1	9	7	8	9	116	79.5
3	1	1	9	7	8	9	113	77.4
4	7	1	9	7	8	9	112	76.7
5	21	2	9	6	8	9	107	73.3
6	4	1	9	6	8	9	106	72.6
7	2	1	9	6	8	9	103	70.5
8	20	1	9	6	7.5	9	101	69.2
9	23	1	9	6	7	9	101	69.2
10	11	1	9	6	7	8.75	98	67.1

3-5 years duration (n=115)

Rank	Question	Range: Minimum	Range: Maximum	Percentiles: 25th	Percentiles: 50th	Percentiles: 75th	N who ranked ≥7	% Agreement
1	19	1	9	7	8	9	92	80.0
2	9	1	9	6	8	9	85	73.9
3	21	1	9	6	8	9	84	73.0
4	2	1	9	6	7	9	83	72.2
5	20	1	9	6	8	9	81	70.4
6	4	1	9	6	8	9	79	68.7
7	3	1	9	6	8	8.5	78	67.8
8	6	1	9	6	7	8	77	67.0
9	23	1	9	6	8	9	77	67.0
10	7	1	9	6	7	9	75	65.2

5-9 years duration (n=126)

Rank	Question	Range: Minimum	Range: Maximum	Percentiles: 25th	Percentiles: 50th	Percentiles: 75th	N who ranked ≥7	% Agreement
1	2	1	9	7	8	9	95	75.4
1	19	3	9	7	8	9	95	75.4
3	9	1	9	6	8	9	93	73.8
4	3	1	9	6	8	9	92	73.0
5	21	1	9	6	8	8	91	72.2
6	4	1	9	6	8	9	88	69.8
7	23	1	9	6	8	9	88	69.8
8	7	1	9	6	8	8	86	68.3
9	6	1	9	6	7.5	9	86	68.3
10	16	1	9	6	7	8	82	65.1

9+ years duration (n=103)

Rank	Question	Range: Minimum	Range: Maximum	Percentiles: 25th	Percentiles: 50th	Percentiles: 75th	N who ranked ≥7	% Agreement
1	19	3	9	7	8	9	85	82.5
2	4	3	9	7	8	9	83	80.6
3	9	2	9	7	8	9	82	79.6
4	2	1	9	6	8	9	74	71.8
5	20	1	9	6	8	9	74	71.8
6	23	1	9	6	8	9	73	70.9
7	11	1	9	6	7	8	72	69.9
8	6	1	9	6	8	8.75	71	68.9
9	21	1	9	6	7.5	9	71	68.9
10	7	2	9	6	8	8	70	68.0

Table 8. Top 10 by Education Level

Level 1 - 2 (n=64)

		Range		Percentiles				
Rank	Question	Minimum	Maximum	25th	50th	75th	N who	%
1	19	3	9	7	8	9	54	84.4
2	9	2	9	7	8	9	54	84.4
3	21	1	9	7	8	9	54	84.4
4	23	4	9	7	8	9	50	78.1
5	11	1	9	7	7	9	50	78.1
6	2	1	9	7	8	9	49	76.6
7	16	4	9	6.75	8	9	48	75.0
8	10	3	9	6.75	8	9	48	75.0
9	20	1	9	6.75	7	9	48	75.0
10	4	2	9	6	8	9	47	73.4

Level 3 - 4 (n=135)

		Range		Percentiles				
Rank	Question	Minimum	Maximum	25th	50th	75th	N who ranked ≥7	% Agreement
1	19	3	9	7	8	9	114	84.4
2	21	1	9	7	8	9	110	81.5
3	2	2	9	7	8	9	103	76.3
4	9	1	9	7	8	9	103	76.3
5	4	1	9	7	8	9	102	75.6
5	20	1	9	7	8	9	102	75.6
7	23	1	9	6	8	9	100	74.1
8	7	1	9	6	8	9	99	73.3
9	8	1	9	6	8	9	98	72.6
10	1	1	9	6	8	9	97	71.9

Level 5 - 6 (n=128)

		Range		Percentiles				
Rank	Question	Minimum	Maximum	25th	50th	75th	N who ranked ≥7	% Agreement
1	19	3	9	7	8	9	100	78.1
2	9	2	9	6	8	9	94	73.4
3	3	2	9	6	8	9	93	72.7
4	4	2	9	6	8	9	92	71.9
5	2	1	9	6	7	9	92	71.9
6	7	1	9	6	7.5	9	90	70.3
7	1	1	9	6	8	9	84	65.6
8	6	1	9	6	7	8	84	65.6
9	21	1	9	6	7	8	82	64.1
10	13	2	9	6	7	8	80	62.5

Level 7 - 8 (n=155)

		Range		Percentiles				
Rank	Question	Minimum	Maximum	25th	50th	75th	N who ranked ≥7	% Agreement
1	9	1	9	7	8	9	120	77.4
2	19	1	9	6	8	9	113	72.9
3	4	1	9	6	8	9	106	68.4
4	21	1	9	6	7	9	104	67.1
5	2	1	9	6	8	9	103	66.5
6	23	1	9	6	7	8	103	66.5
7	1	1	9	6	7	8	101	65.2
8	7	1	9	6	7	8.5	100	64.5
9	3	1	9	6	7	8	97	62.6
10	6	1	9	6	7	8	96	61.9

Table 9. Top 10 by Living Arrangements

Living at Home Independently (n=331)								
		Range		Percentile				
Rank	Question	Minimum	Maximum	25th	50th	75th	N who ranked ≥7	% Agreement
1	19	1	9	7	8	9	261	78.9
2	9	1	9	7	8	9	259	78.2
3	21	1	9	6	8	9	247	74.6
4	3	1	9	6	8	9	236	71.3
5	4	1	9	6	8	9	235	71.0
6	1	1	9	6	8	9	231	69.8
6	2	1	9	6	8	9	231	69.8
8	7	1	9	6	8	9	229	69.2
9	23	1	9	6	7	9	228	68.9
10	20	1	9	6	7	9	223	67.4
**Living at Home Supported by Family (n=123)**								
		Range		Percentile				
Rank	Question	Minimum	Maximum	25th	50th	75th	N who ranked ≥7	% Agreement
1	19	2	9	7	8	9	96	78.0
2	6	1	9	7	8	9	95	77.2
3	2	1	9	7	8	9	94	76.4
4	4	1	9	7	8	9	93	75.6
5	9	1	9	6	8	9	89	72.4
6	16	1	9	6	8	8	86	69.9
7	1	1	9	6	7	9	84	68.3
8	23	1	9	6	7	8.5	84	68.3
9	7	1	9	6	7	8	82	66.7
10	21	1	9	6	8	8	80	65.0
**Needing Carers or in Supported Home (n=25)**								
		Range		Percentile				
Rank	Question	Minimum	Maximum	25th	50th	75th	N who ranked ≥7	% Agreement
1	19	5	9	7	8	9	23	92.0
2	2	2	9	7	8	9	23	92.0
3	7	5	9	7	9	9	22	88.0
4	5	3	9	7	9	9	21	84.0
5	9	4	9	8	8	9	21	84.0
6	23	1	9	7	8	9	21	84.0
7	16	1	9	7	7	9	21	84.0
8	4	4	9	8	8	9	20	80.0
9	15	4	9	7	8	9	20	80.0

Table 10. Top 10 by Local Institute

UL Summary (n=303)								
		Range		Percentile				
Rank	Question	Minimum	Maximum	25th	50th	75th	N who ranked ≥7	% Agreement
1	2	2	9	8	8	9	275	90.8
2	9	4	9	7	8	9	268	88.4
3	1	1	9	7	8	9	266	87.8
4	4	1	9	7	8	9	263	86.8
5	7	1	9	7	8	9	258	85.1
6	19	3	9	7	8	9	257	84.8
7	21	1	9	7	8	9	253	83.5
7	23	1	9	7	8	9	253	83.5
9	15	1	9	7	8	9	250	82.5
10	14	2	9	7	8	9	247	81.5

EKUT Summary (n=176)								
		Range		Percentile				
Rank	Question	Minimum	Maximum	25th	50th	75th	N who ranked ≥7	% Agreement
1	19	1	9	7	8	9	141	80.1
2	4	1	9	7	8	9	134	76.1
3	7	1	9	7	8	9	133	75.6
4	9	1	9	6	8	9	130	73.9
5	21	1	9	6	8	9	127	72.2
5	23	1	9	6	8	9	127	72.2
7	20	1	9	6	8	9	126	71.6
8	2	1	9	6	8	9	124	70.5
9	13	2	9	6	8	9	122	69.3
10	1	1	9	6	8	9	114	64.8

UOXF Sumary (n=400)								
		Range		Percentile				
Rank	Question	Minimum	Maximum	25th	50th	75th	N who ranked ≥7	% Agreement
1	19	1	9	6	8	9	297	74.3
1	9	1	9	6	8	9	297	74.3
3	21	1	9	6	7.5	8	293	73.3
4	2	1	9	6	8	9	291	72.8
5	3	1	9	6	8	9	274	68.5
6	6	1	9	6	7	9	274	68.5
7	7	1	9	6	7	8	271	67.8
8	4	1	9	6	7.5	9	270	67.5
9	16	1	9	6	7	8	268	67.0
10	1	1	9	6	7	9	265	66.3

Table 11. Top 10 Priorities by Gender

Male (n=312)								
		Range		Percentiles				
Rank	Question Number	Minimum	Maximum	25th	50th (Median)	75th	N who ranked ≥7	% Agreement
1	19	1	9	7	8	9	246	78.8
2	9	1	9	7	8	9	236	75.6
3	2	1	9	6	8	9	224	71.8
4	1	1	9	6	8	9	218	69.9
5	4	1	9	6	8	9	217	69.6
6	7	1	9	6	7.5	9	216	69.2
7	21	1	9	6	7.5	9	212	67.9
8	3	1	9	6	7	8	207	66.3
9	23	1	9	6	7	9	204	65.4
10	20	1	9	6	7	9	203	65.1

Female (n=192)								
		Range		Percentiles				
Rank	Question Number	Minimum	Maximum	25th	50th (Median)	75th	N who ranked ≥7	% Agreement
1	19	1	9	7	8	9	156	81.3
2	9	1	9	7	8	9	153	79.7
3	21	1	9	7	8	9	151	78.6
4	23	1	9	7	8	9	148	77.1
5	4	1	9	7	8	9	147	76.6
6	2	1	9	6.75	8	9	144	75.0
7	6	1	9	6	8	9	143	74.5
8	7	1	9	6	8	9	137	71.4
9	3	1	9	6	8	9	134	69.8
10	20	1	9	6	8	9	134	69.8

Table 12. Top 10 by Economic Status

Above Poverty Line (n=372)								
		Range		Percentile				
Rank	Question	Minimum	Maximum	25th	50th	75th	N who ranked ≥7	% Agreement
1	19	1	9	7	8	9	291	78.2
2	9	1	9	7	8	9	287	77.2
3	4	1	9	6	8	9	272	73.1
4	21	1	9	6	8	9	268	72.0
5	2	1	9	6	8	9	263	70.7
6	3	1	9	6	8	9	255	68.5
6	7	1	9	6	8	9	255	68.5
8	6	1	9	6	7	8.25	252	67.7
9	23	1	9	6	7	9	248	66.7
10	1	1	9	6	7	9	247	66.4
**Below Poverty Line (n=73)**								
		Range		Percentile				
Rank	Question	Minimum	Maximum	25th	50th	75th	N who ranked ≥7	% Agreement
1	19	3	9	7	8	9	65	89.0
2	2	1	9	7	8	9	60	82.2
3	9	2	9	7	8	9	58	79.5
4	1	2	9	7	8	9	55	75.3
5	23	1	9	7	8	9	55	75.3
6	7	1	9	6	8	9	53	72.6
6	21	1	9	6	8	9	53	72.6
8	20	1	9	6	7	9	53	72.6
9	16	1	9	6	8	9	52	71.2
10	4	1	9	6	8	9	51	69.9

Table 13. Top 10 by Ethnicity Groups

BAME (n=11)								
		Range		Percentiles				
Rank	Question	Minimum	Maximum	25th	50th	75th	N who ranked ≥7	% Agreement
1	6	1	9	5	8	9	10	90.9
2	4	1	9	5	8	9	9	81.8
3	18	1	9	5	8	9	9	81.8
4	3	1	9	5	8	9	8	72.7
5	5	3	9	5	8	9	8	72.7
6	10	1	9	5	8	9	8	72.7
7	16	4	9	5	8	9	8	72.7
8	23	6	9	5	8	9	8	72.7
9	2	4	9	5	8	9	7	63.6
10	8	5	9	5	8	9	7	63.6
**White (n=217)**								
		Range		Percentiles				
Rank	Question	Minimum	Maximum	25th	50th	75th	N who ranked ≥7	% Agreement
1	9	1	9	7	8	9	163	75.1
2	19	1	9	6	8	9	162	74.7
3	21	1	9	6	8	8	153	70.5
4	6	1	9	6	8	9	152	70.0
5	2	1	9	6	7	9	149	68.7
6	3	1	9	6	8	9	146	67.3
7	4	1	9	6	8	8	141	65.0
8	1	1	9	6	7	9	140	64.5
9	16	1	9	5	7	8	137	63.1
10	11	1	9	6	7	8	133	61.3

Kappa Tables

Table 14. Kappa Agreement between Disease Duration Groups

Symmetric Measures				
Comparison	Kappa Value	Asymptotic Standard Error *a*	Approximate T *b*	Approximate Significance
0-3 years vs 3-5 years	0.682	0.146	3.546	0.000
0-3 years v 5-9 years	0.524	0.170	2.720	0.007
0-3 years v 9+ years	0.841	0.108	4.371	0.000
3-5 years v 5-9 years	0.841	0.108	4.371	0.000
3-5 years v 9+ years	0.841	0.108	4.371	0.000
5-9 years v 9+ years	0.682	0.146	3.546	0.000
a Not assuming the null hypothesis.				
b Using the asymptotic standard error assuming the null hypothesis.				

Table 15. Kappa Agreement between Education Levels

Symmetric Measures				
Comparison	Kappa	Asymptotic Standard Error *a*	Approximate T *b*	Approximate Significance
Level 1 - 2 vs Level 3 - 4	0.524	0.170	2.720	0.007
Level 1 - 2 vs Level 5 - 6	0.206	0.192	1.070	0.285
Level 1 - 2 vs Level 7 - 8	0.365	0.185	1.895	0.058
Level 3 - 4 vs Level 5 - 6	0.524	0.170	2.720	0.007
Level 3 - 4 vs Level 7 - 8	0.682	0.146	3.546	0.000
Level 5 - 6 vs Level 7 - 8	0.841	0.108	4.371	0.000
a Not assuming the null hypothesis.				
b Using the asymptotic standard error assuming the null hypothesis.				

Table 16. Kappa test of Agreement by Living Arrangement (grouped)

Symmetric Measures				
Comparison	Kappa	Asymptotic Standard Error *a*	Approximate T *b*	Approximate Significance
Independently vs Supported by Family	0.682	0.146	3.546	0.000
Independently vs Carers/Supported Home	0.524	0.170	2.720	0.007
Supported by Family vs Carers/Supported Home	0.682	0.146	3.546	0.000
N of Valid Cases	27			
a Not assuming the null hypothesis.				
b Using the asymptotic standard error assuming the null hypothesis.				

Table 17. Kappa Test for Agreement between Local Institutes

Measure of Agreement				
Comparison	Kappa	Asymptotic Standard Error *a*	Approximate T *b*	Approximate Significance
UL * EKUT	0.682	0.146	3.546	0
UL * UOXF	0.524	0.17	2.72	0.007
EKUT * UOXF	0.524	0.17	2.72	0.007
a Not assuming the null hypothesis.				
b Using the asymptotic standard error assuming the null hypothesis.				

Table 18. Kappa for Gender

Symmetric Measures

		Value	Asymptotic Standard Error[a]	Approximate T[b]	Approximate Significance
Measure of Agreement	Kappa	.841	.108	4.371	.000
N of Valid Cases		27			

a. Not assuming the null hypothesis.

b. Using the asymptotic standard error assuming the null hypothesis.

Table 19. Kappa for Economic Status

Symmetric Measures

		Value	Asymptotic Standard Error[a]	Approximate T[b]	Approximate Significance
Measure of Agreement	Kappa	.682	.146	3.546	.000
N of Valid Cases		27			

a. Not assuming the null hypothesis.

b. Using the asymptotic standard error assuming the null hypothesis.

Table 20. Kappa test between HCP and PwP

Symmetric Measures

		Value	Asymptotic Standard Error[a]	Approximate T[b]	Approximate Significance
Measure of Agreement	Kappa	.682	.146	3.546	.000
N of Valid Cases		27			

a. Not assuming the null hypothesis.

b. Using the asymptotic standard error assuming the null hypothesis.

Table 21. Kappa test with Deane et al Top 10 by Focus group

Symmetric Measures

		Value	Asymptotic Standard Error[a]	Approximate T[b]	Approximate Significance
Measure of Agreement	Kappa	.206	.192	1.070	.285
N of Valid Cases		27			

a. Not assuming the null hypothesis.

b. Using the asymptotic standard error assuming the null hypothesis.

Table 22. Kappa test with Deane et al by interim ranking

Symmetric Measures

		Value	Asymptotic Standard Error[a]	Approximate T[b]	Approximate Significance
Measure of Agreement	Kappa	.524	.170	2.720	.007
N of Valid Cases		27			

a. Not assuming the null hypothesis.

b. Using the asymptotic standard error assuming the null hypothesis.

Distribution Analyses

Table 23. Distribution Comparisons of each question by subgroup

		Significance (p-value)						
**Question Number**	**Question**	**HCP vs PwP (MWW)**	**Disease Duration (KW)**	**Education Level (KW)**	**Living Arrangements (KW)**	**Local Institute (KW)**	**Gender (MWW)**	**Economic Status (MWW)**
**1**	What treatments are helpful in reducing tremor in people with Parkinson’s?	0.001	0.305	0.027	0.591	0.000	0.960	0.113
**2**	What treatments are helpful for reducing balance problems and falls in people with Parkinson’s?	0.000	0.995	0.043	0.088	0.000	0.120	0.268
**3**	Is it possible to identify different types of Parkinson’s, e.g., tremor dominant? And can we tailor treatments best according to these different types?	0.794	0.087	0.403	0.321	0.079	0.027	0.888
**4**	What treatments would ensure the medications were equally effective each day (prevented/managed wearing off, variability, on/off states) in people with Parkinson’s?	0.275	0.040	0.752	0.186	0.000	0.023	0.907
**5**	Would the monitoring of dopamine levels in the body (e.g., with blood tests) be helpful in determining medication timing and amount (dose)?	0.084	0.156	0.161	0.001	0.000	0.022	0.043
**6**	What is helpful for improving the quality of sleep in people with Parkinson’s?	0.703	0.987	0.009	0.212	0.035	0.001	0.154

**7**	What best treats mild cognitive problems such as memory loss, lack of concentration, indecision and slowed thinking in people with Parkinson's?	0.091	0.300	0.502	0.012	0.000	0.174	0.499
**8**	What treatments are helpful in reducing urinary problems (urgency, irritable bladder, incontinence) in people with Parkinson's?	0.592	0.953	0.011	0.825	0.000	0.413	0.950
**9**	What drug treatments are best for the different stages of Parkinson's?	0.248	0.728	0.529	0.059	0.001	0.201	0.756
**10**	What approaches are helpful for reducing stress and anxiety in people with Parkinson's?	0.008	0.387	0.000	0.097	0.000	0.008	0.288
**11**	What treatments are helpful for reducing dyskinesias (involuntary movements, which are a side effect of some medications) in people with Parkinson's?	0.015	0.653	0.004	0.079	0.000	0.001	0.426
**12**	What best treats dementia in people with Parkinson's?	0.041	0.103	0.027	0.249	0.000	0.106	0.122
**13**	What interventions are effective for reducing or managing unexplained fatigue in people with Parkinson's?	0.285	0.502	0.180	0.356	0.000	0.887	0.932
**14**	What best helps prevent or reduce freezing (of gait and in general) in people with Parkinson's?	0.002	0.411	0.067	0.402	0.000	0.049	0.322

**15**	What treatments are helpful for swallowing problems (dysphagia) in people with Parkinson's?	0.000	0.331	0.009	0.043	0.000	0.014	0.934
**16**	What is the best method of monitoring a person with Parkinson's response to treatments?	0.602	0.629	0.000	0.053	0.004	0.189	0.034
**17**	What training, techniques or aids are needed for hospital staff, to make sure patients with Parkinson's get their medications correctly and on time?	0.007	0.603	0.000	0.002	0.000	0.001	0.090
**18**	What treatments are helpful in reducing bowel problems (constipation, incontinence) in people with Parkinson's?	0.224	0.650	0.000	0.468	0.000	0.125	0.411
**19**	What is the best type and dose of exercise (physiotherapy) for improving muscle strength, flexibility, fitness, balance and function in people with Parkinson's?	0.072	0.439	0.026	0.439	0.000	0.166	0.687
**20**	Can medications be developed to allow fewer doses per day for people with Parkinson's? (For example combinations of medications in one pill, slow release pills)	0.347	0.106	0.000	0.071	0.000	0.221	0.273
**21**	What helps improve the dexterity (fine motor skills or coordination of small muscle movements) of people with Parkinson's so they can do up buttons, use computers, phones, remote controls etc?	0.337	0.739	0.009	0.066	0.004	0.005	0.540
**22**	What treatments are effective in reducing hallucinations (including vivid dreams) in people with Parkinson's?	0.000	0.170	0.000	0.322	0.000	0.225	0.379

**23**	What is the best treatment for stiffness (rigidity) in people with Parkinson's?	0.185	0.680	0.033	0.137	0.000	0.016	0.083
**24**	At which stage of Parkinson's is deep brain stimulation (a surgical treatment that involves implanting a 'brain pacemaker' that sends signals to specific parts of the brain) most helpful?	0.423	0.691	0.383	0.090	0.000	0.853	0.152
**25**	What training to improve knowledge and skills do informal carers (family and friends) need in order to best care for people with Parkinson's?	0.000	0.417	0.000	0.884	0.000	0.297	0.467
**26**	What is the best treatment for pain in people with Parkinson's?	0.020	0.258	0.002	0.827	0.000	0.001	0.116
**27**	What speech therapy techniques are helpful for communication problems in people with Parkinson's?	0.001	0.731	0.001	0.337	0.000	0.421	0.813

Table 24. Pairwise comparison of significant KW Disease Duration for Question 4.

Pairwise Comparisons of Duration of Disease

Sample 1-Sample 2	Test Statistic	Std. Error	Std. Test Statistic	Sig.	Adj. Sig.[a]
0 - 3 years-5 - 9 years	-1.548	16.698	-.093	.926	1.000
0 - 3 years-3 - 5 years	-19.389	17.121	-1.132	.257	1.000
0 - 3 years-9+ years	-46.026	17.671	-2.605	.009	.055
5 - 9 years-3 - 5 years	17.841	17.710	1.007	.314	1.000
5 - 9 years-9+ years	-44.478	18.241	-2.438	.015	.089
3 - 5 years-9+ years	-26.638	18.630	-1.430	.153	.917

Each row tests the null hypothesis that the Sample 1 and Sample 2 distributions are the same.
Asymptotic significances (2-sided tests) are displayed. The significance level is .050.

a. Significance values have been adjusted by the Bonferroni correction for multiple tests.

Table 25. Pairwise comparison of significant KW Education Level for Question 1.

Pairwise Comparisons of What is their highest education level?

Sample 1-Sample 2	Test Statistic	Std. Error	Std. Test Statistic	Sig.	Adj. Sig.[a]
Level 7 - 8-Level 5 - 6	33.346	16.224	2.055	.040	.239
Level 7 - 8-Level 1 - 2	42.944	20.184	2.128	.033	.200
Level 7 - 8-Level 3 - 4	43.192	15.992	2.701	.007	.041
Level 5 - 6-Level 1 - 2	9.598	20.796	.462	.644	1.000
Level 5 - 6-Level 3 - 4	9.846	16.759	.587	.557	1.000
Level 1 - 2-Level 3 - 4	-.248	20.616	-.012	.990	1.000

Each row tests the null hypothesis that the Sample 1 and Sample 2 distributions are the same.
Asymptotic significances (2-sided tests) are displayed. The significance level is .050.

a. Significance values have been adjusted by the Bonferroni correction for multiple tests.

Table 26. Pairwise comparison of significant KW Education Level for Question 2.

Pairwise Comparisons of What is their highest education level?

Sample 1-Sample 2	Test Statistic	Std. Error	Std. Test Statistic	Sig.	Adj. Sig.[a]
Level 7 - 8-Level 5 - 6	9.144	16.210	.564	.573	1.000
Level 7 - 8-Level 3 - 4	34.072	15.978	2.132	.033	.198
Level 7 - 8-Level 1 - 2	47.371	20.166	2.349	.019	.113
Level 5 - 6-Level 3 - 4	24.928	16.744	1.489	.137	.819
Level 5 - 6-Level 1 - 2	38.227	20.779	1.840	.066	.395
Level 3 - 4-Level 1 - 2	13.298	20.598	.646	.519	1.000

Each row tests the null hypothesis that the Sample 1 and Sample 2 distributions are the same.
Asymptotic significances (2-sided tests) are displayed. The significance level is .050.

a. Significance values have been adjusted by the Bonferroni correction for multiple tests.

Table 27. Pairwise comparison of significant KW Education Level for Question 6.

Pairwise Comparisons of What is their highest education level?

Sample 1-Sample 2	Test Statistic	Std. Error	Std. Test Statistic	Sig.	Adj. Sig.[a]
Level 7 - 8-Level 5 - 6	15.056	16.313	.923	.356	1.000
Level 7 - 8-Level 3 - 4	45.459	16.079	2.827	.005	.028
Level 7 - 8-Level 1 - 2	52.903	20.294	2.607	.009	.055
Level 5 - 6-Level 3 - 4	30.403	16.850	1.804	.071	.427
Level 5 - 6-Level 1 - 2	37.848	20.910	1.810	.070	.422
Level 3 - 4-Level 1 - 2	7.445	20.729	.359	.719	1.000

Each row tests the null hypothesis that the Sample 1 and Sample 2 distributions are the same.
Asymptotic significances (2-sided tests) are displayed. The significance level is .050.

a. Significance values have been adjusted by the Bonferroni correction for multiple tests.

Table 28. Pairwise comparison of significant KW Education Level for Question 8.

Pairwise Comparisons of What is their highest education level?

Sample 1–Sample 2	Test Statistic	Std. Error	Std. Test Statistic	Sig.	Adj. Sig.[a]
Level 5 – 6–Level 7 – 8	-5.576	16.338	-.341	.733	1.000
Level 5 – 6–Level 1 – 2	41.242	20.943	1.969	.049	.294
Level 5 – 6–Level 3 – 4	46.542	16.877	2.758	.006	.035
Level 7 – 8–Level 1 – 2	35.667	20.326	1.755	.079	.476
Level 7 – 8–Level 3 – 4	40.966	16.104	2.544	.011	.066
Level 1 – 2–Level 3 – 4	-5.300	20.761	-.255	.799	1.000

Each row tests the null hypothesis that the Sample 1 and Sample 2 distributions are the same.
Asymptotic significances (2-sided tests) are displayed. The significance level is .050.

a. Significance values have been adjusted by the Bonferroni correction for multiple tests.

Table 29. Pairwise comparison of significant KW Education Level for Question 10.

Pairwise Comparisons of What is their highest education level?

Sample 1–Sample 2	Test Statistic	Std. Error	Std. Test Statistic	Sig.	Adj. Sig.[a]
Level 7 – 8–Level 5 – 6	26.177	16.431	1.593	.111	.667
Level 7 – 8–Level 3 – 4	44.349	16.196	2.738	.006	.037
Level 7 – 8–Level 1 – 2	88.634	20.441	4.336	.000	.000
Level 5 – 6–Level 3 – 4	18.172	16.972	1.071	.284	1.000
Level 5 – 6–Level 1 – 2	62.457	21.062	2.965	.003	.018
Level 3 – 4–Level 1 – 2	44.285	20.879	2.121	.034	.204

Each row tests the null hypothesis that the Sample 1 and Sample 2 distributions are the same.
Asymptotic significances (2-sided tests) are displayed. The significance level is .050.

a. Significance values have been adjusted by the Bonferroni correction for multiple tests.

Table 30. Pairwise comparison of significant KW Education Level for Question 11.

Pairwise Comparisons of What is their highest education level?

Sample 1–Sample 2	Test Statistic	Std. Error	Std. Test Statistic	Sig.	Adj. Sig.[a]
Level 7 – 8–Level 5 – 6	20.802	16.359	1.272	.204	1.000
Level 7 – 8–Level 3 – 4	48.662	16.126	3.018	.003	.015
Level 7 – 8–Level 1 – 2	59.869	20.352	2.942	.003	.020
Level 5 – 6–Level 3 – 4	27.860	16.899	1.649	.099	.595
Level 5 – 6–Level 1 – 2	39.066	20.970	1.863	.062	.375
Level 3 – 4–Level 1 – 2	11.206	20.788	.539	.590	1.000

Each row tests the null hypothesis that the Sample 1 and Sample 2 distributions are the same.
Asymptotic significances (2-sided tests) are displayed. The significance level is .050.

a. Significance values have been adjusted by the Bonferroni correction for multiple tests.

Table 31. Pairwise comparison of significant KW Education Level for Question 12.

Pairwise Comparisons of What is their highest education level?

Sample 1–Sample 2	Test Statistic	Std. Error	Std. Test Statistic	Sig.	Adj. Sig.[a]
Level 5 – 6–Level 7 – 8	-20.583	16.349	-1.259	.208	1.000
Level 5 – 6–Level 3 – 4	43.937	16.888	2.602	.009	.056
Level 5 – 6–Level 1 – 2	50.020	20.956	2.387	.017	.102
Level 7 – 8–Level 3 – 4	23.354	16.115	1.449	.147	.884
Level 7 – 8–Level 1 – 2	29.437	20.339	1.447	.148	.887
Level 3 – 4–Level 1 – 2	6.083	20.775	.293	.770	1.000

Each row tests the null hypothesis that the Sample 1 and Sample 2 distributions are the same.
Asymptotic significances (2-sided tests) are displayed. The significance level is .050.

a. Significance values have been adjusted by the Bonferroni correction for multiple tests.

Table 32. Pairwise comparison of significant KW Education Level for Question 15.

Pairwise Comparisons of What is their highest education level?

Sample 1-Sample 2	Test Statistic	Std. Error	Std. Test Statistic	Sig.	Adj. Sig.[a]
Level 7 - 8-Level 5 - 6	15.901	16.422	.968	.333	1.000
Level 7 - 8-Level 3 - 4	43.749	16.187	2.703	.007	.041
Level 7 - 8-Level 1 - 2	56.421	20.430	2.762	.006	.035
Level 5 - 6-Level 3 - 4	27.848	16.963	1.642	.101	.604
Level 5 - 6-Level 1 - 2	40.520	21.051	1.925	.054	.325
Level 3 - 4-Level 1 - 2	12.672	20.868	.607	.544	1.000

Each row tests the null hypothesis that the Sample 1 and Sample 2 distributions are the same.
Asymptotic significances (2-sided tests) are displayed. The significance level is .050.

a. Significance values have been adjusted by the Bonferroni correction for multiple tests.

Table 33. Pairwise comparison of significant KW Education Level for Question 16.

Pairwise Comparisons of What is their highest education level?

Sample 1-Sample 2	Test Statistic	Std. Error	Std. Test Statistic	Sig.	Adj. Sig.[a]
Level 7 - 8-Level 5 - 6	18.679	16.379	1.140	.254	1.000
Level 7 - 8-Level 3 - 4	41.464	16.145	2.568	.010	.061
Level 7 - 8-Level 1 - 2	81.487	20.376	3.999	.000	.000
Level 5 - 6-Level 3 - 4	22.785	16.919	1.347	.178	1.000
Level 5 - 6-Level 1 - 2	62.809	20.995	2.992	.003	.017
Level 3 - 4-Level 1 - 2	40.023	20.813	1.923	.054	.327

Each row tests the null hypothesis that the Sample 1 and Sample 2 distributions are the same.
Asymptotic significances (2-sided tests) are displayed. The significance level is .050.

a. Significance values have been adjusted by the Bonferroni correction for multiple tests.

Table 34. Pairwise comparison of significant KW Education Level for Question 17.

Pairwise Comparisons of What is their highest education level?

Sample 1-Sample 2	Test Statistic	Std. Error	Std. Test Statistic	Sig.	Adj. Sig.[a]
Level 7 - 8-Level 5 - 6	54.895	16.464	3.334	.001	.005
Level 7 - 8-Level 3 - 4	68.367	16.229	4.213	.000	.000
Level 7 - 8-Level 1 - 2	98.266	20.483	4.798	.000	.000
Level 5 - 6-Level 3 - 4	13.471	17.007	.792	.428	1.000
Level 5 - 6-Level 1 - 2	43.371	21.104	2.055	.040	.239
Level 3 - 4-Level 1 - 2	29.900	20.921	1.429	.153	.918

Each row tests the null hypothesis that the Sample 1 and Sample 2 distributions are the same.
Asymptotic significances (2-sided tests) are displayed. The significance level is .050.

a. Significance values have been adjusted by the Bonferroni correction for multiple tests.

Table 35. Pairwise comparison of significant KW Education Level for Question 18.

Pairwise Comparisons of What is their highest education level?

Sample 1-Sample 2	Test Statistic	Std. Error	Std. Test Statistic	Sig.	Adj. Sig.[a]
Level 7 - 8-Level 5 - 6	41.617	16.381	2.541	.011	.066
Level 7 - 8-Level 3 - 4	53.936	16.147	3.340	.001	.005
Level 7 - 8-Level 1 - 2	76.063	20.379	3.732	.000	.001
Level 5 - 6-Level 3 - 4	12.318	16.921	.728	.467	1.000
Level 5 - 6-Level 1 - 2	34.445	20.998	1.640	.101	.605
Level 3 - 4-Level 1 - 2	22.127	20.815	1.063	.288	1.000

Each row tests the null hypothesis that the Sample 1 and Sample 2 distributions are the same.
Asymptotic significances (2-sided tests) are displayed. The significance level is .050.

a. Significance values have been adjusted by the Bonferroni correction for multiple tests.

Table 36. Pairwise comparison of significant KW Education Level for Question 19.

Pairwise Comparisons of What is their highest education level?

Sample 1-Sample 2	Test Statistic	Std. Error	Std. Test Statistic	Sig.	Adj. Sig.[a]
Level 7 - 8-Level 1 - 2	34.781	19.958	1.743	.081	.488
Level 7 - 8-Level 5 - 6	39.973	16.042	2.492	.013	.076
Level 7 - 8-Level 3 - 4	41.481	15.813	2.623	.009	.052
Level 1 - 2-Level 5 - 6	-5.191	20.563	-.252	.801	1.000
Level 1 - 2-Level 3 - 4	-6.700	20.385	-.329	.742	1.000
Level 5 - 6-Level 3 - 4	1.509	16.571	.091	.927	1.000

Each row tests the null hypothesis that the Sample 1 and Sample 2 distributions are the same.
Asymptotic significances (2-sided tests) are displayed. The significance level is .050.

a. Significance values have been adjusted by the Bonferroni correction for multiple tests.

Table 37. Pairwise comparison of significant KW Education Level for Question 20.

Pairwise Comparisons of What is their highest education level?

Sample 1-Sample 2	Test Statistic	Std. Error	Std. Test Statistic	Sig.	Adj. Sig.[a]
Level 7 - 8-Level 5 - 6	9.380	16.279	.576	.564	1.000
Level 7 - 8-Level 1 - 2	46.493	20.253	2.296	.022	.130
Level 7 - 8-Level 3 - 4	61.676	16.047	3.844	.000	.001
Level 5 - 6-Level 1 - 2	37.113	20.868	1.779	.075	.452
Level 5 - 6-Level 3 - 4	52.296	16.816	3.110	.002	.011
Level 1 - 2-Level 3 - 4	-15.182	20.686	-.734	.463	1.000

Each row tests the null hypothesis that the Sample 1 and Sample 2 distributions are the same.
Asymptotic significances (2-sided tests) are displayed. The significance level is .050.

a. Significance values have been adjusted by the Bonferroni correction for multiple tests.

Table 38. Pairwise comparison of significant KW Education Level for Question 21.

Pairwise Comparisons of What is their highest education level?

Sample 1-Sample 2	Test Statistic	Std. Error	Std. Test Statistic	Sig.	Adj. Sig.[a]
Level 5 - 6-Level 7 - 8	-3.987	16.228	-.246	.806	1.000
Level 5 - 6-Level 3 - 4	40.673	16.762	2.426	.015	.091
Level 5 - 6-Level 1 - 2	52.379	20.801	2.518	.012	.071
Level 7 - 8-Level 3 - 4	36.686	15.995	2.294	.022	.131
Level 7 - 8-Level 1 - 2	48.392	20.188	2.397	.017	.099
Level 3 - 4-Level 1 - 2	11.706	20.621	.568	.570	1.000

Each row tests the null hypothesis that the Sample 1 and Sample 2 distributions are the same.
Asymptotic significances (2-sided tests) are displayed. The significance level is .050.

a. Significance values have been adjusted by the Bonferroni correction for multiple tests.

Table 39. Pairwise comparison of significant KW Education Level for Question 22.

Pairwise Comparisons of What is their highest education level?

Sample 1-Sample 2	Test Statistic	Std. Error	Std. Test Statistic	Sig.	Adj. Sig.[a]
Level 7 - 8-Level 5 - 6	8.198	16.498	.497	.619	1.000
Level 7 - 8-Level 3 - 4	48.578	16.263	2.987	.003	.017
Level 7 - 8-Level 1 - 2	82.558	20.525	4.022	.000	.000
Level 5 - 6-Level 3 - 4	40.380	17.042	2.369	.018	.107
Level 5 - 6-Level 1 - 2	74.359	21.148	3.516	.000	.003
Level 3 - 4-Level 1 - 2	33.979	20.965	1.621	.105	.630

Each row tests the null hypothesis that the Sample 1 and Sample 2 distributions are the same.
Asymptotic significances (2-sided tests) are displayed. The significance level is .050.

a. Significance values have been adjusted by the Bonferroni correction for multiple tests.

Table 40. Pairwise comparison of significant KW Education Level for Question 23.

Pairwise Comparisons of What is their highest education level?

Sample 1-Sample 2	Test Statistic	Std. Error	Std. Test Statistic	Sig.	Adj. Sig.[a]
Level 5 - 6-Level 7 - 8	-2.274	16.275	-.140	.889	1.000
Level 5 - 6-Level 3 - 4	23.389	16.811	1.391	.164	.985
Level 5 - 6-Level 1 - 2	54.359	20.862	2.606	.009	.055
Level 7 - 8-Level 3 - 4	21.115	16.042	1.316	.188	1.000
Level 7 - 8-Level 1 - 2	52.085	20.247	2.572	.010	.061
Level 3 - 4-Level 1 - 2	30.970	20.681	1.498	.134	.805

Each row tests the null hypothesis that the Sample 1 and Sample 2 distributions are the same.
Asymptotic significances (2-sided tests) are displayed. The significance level is .050.

a. Significance values have been adjusted by the Bonferroni correction for multiple tests.

Table 41. Pairwise comparison of significant KW Education Level for Question 25.

Pairwise Comparisons of What is their highest education level?

Sample 1-Sample 2	Test Statistic	Std. Error	Std. Test Statistic	Sig.	Adj. Sig.[a]
Level 7 - 8-Level 5 - 6	39.761	16.439	2.419	.016	.093
Level 7 - 8-Level 3 - 4	65.627	16.204	4.050	.000	.000
Level 7 - 8-Level 1 - 2	69.667	20.451	3.406	.001	.004
Level 5 - 6-Level 3 - 4	25.867	16.981	1.523	.128	.766
Level 5 - 6-Level 1 - 2	29.906	21.072	1.419	.156	.935
Level 3 - 4-Level 1 - 2	4.040	20.889	.193	.847	1.000

Each row tests the null hypothesis that the Sample 1 and Sample 2 distributions are the same.
Asymptotic significances (2-sided tests) are displayed. The significance level is .050.

a. Significance values have been adjusted by the Bonferroni correction for multiple tests.

Table 42. Pairwise comparison of significant KW Education Level for Question 26.

Pairwise Comparisons of What is their highest education level?

Sample 1-Sample 2	Test Statistic	Std. Error	Std. Test Statistic	Sig.	Adj. Sig.[a]
Level 7 - 8-Level 5 - 6	20.230	16.419	1.232	.218	1.000
Level 7 - 8-Level 3 - 4	53.546	16.184	3.309	.001	.006
Level 7 - 8-Level 1 - 2	57.199	20.426	2.800	.005	.031
Level 5 - 6-Level 3 - 4	33.316	16.960	1.964	.049	.297
Level 5 - 6-Level 1 - 2	36.969	21.046	1.757	.079	.474
Level 3 - 4-Level 1 - 2	3.653	20.863	.175	.861	1.000

Each row tests the null hypothesis that the Sample 1 and Sample 2 distributions are the same.
Asymptotic significances (2-sided tests) are displayed. The significance level is .050.

a. Significance values have been adjusted by the Bonferroni correction for multiple tests.

Table 43. Pairwise comparison of significant KW Education Level for Question 27.

Pairwise Comparisons of What is their highest education level?

Sample 1-Sample 2	Test Statistic	Std. Error	Std. Test Statistic	Sig.	Adj. Sig.[a]
Level 7 - 8-Level 5 - 6	27.543	16.440	1.675	.094	.563
Level 7 - 8-Level 3 - 4	51.247	16.205	3.162	.002	.009
Level 7 - 8-Level 1 - 2	71.110	20.453	3.477	.001	.003
Level 5 - 6-Level 3 - 4	23.703	16.982	1.396	.163	.977
Level 5 - 6-Level 1 - 2	43.566	21.074	2.067	.039	.232
Level 3 - 4-Level 1 - 2	19.863	20.891	.951	.342	1.000

Each row tests the null hypothesis that the Sample 1 and Sample 2 distributions are the same.
Asymptotic significances (2-sided tests) are displayed. The significance level is .050.

a. Significance values have been adjusted by the Bonferroni correction for multiple tests.

Table 44. Pairwise comparison of significant KW Living Arrangements for Question 5.

Pairwise Comparisons of What are the living arrangements of the participant?

Sample 1-Sample 2	Test Statistic	Std. Error	Std. Test Statistic	Sig.	Adj. Sig.[a]
Own home (independently)-Own home (supported by family)	-3.475	14.416	-.241	.810	1.000
Own home (independently)-SBC	-106.575	28.316	-3.764	.000	.001
Own home (supported by family)-SBC	-103.100	29.950	-3.442	.001	.002

Each row tests the null hypothesis that the Sample 1 and Sample 2 distributions are the same.
Asymptotic significances (2-sided tests) are displayed. The significance level is .050.

a. Significance values have been adjusted by the Bonferroni correction for multiple ...

Table 45. Pairwise comparison of significant KW Living Arrangements for Question 7.

Pairwise Comparisons of What are the living arrangements of the participant?

Sample 1-Sample 2	Test Statistic	Std. Error	Std. Test Statistic	Sig.	Adj. Sig.[a]
Own home (supported by family)-Own home (independently)	21.187	14.291	1.483	.138	.415
Own home (supported by family)-SBC	-86.875	29.690	-2.926	.003	.010
Own home (independently)-SBC	-65.688	28.070	-2.340	.019	.058

Each row tests the null hypothesis that the Sample 1 and Sample 2 distributions are the same.
Asymptotic significances (2-sided tests) are displayed. The significance level is .050.

a. Significance values have been adjusted by the Bonferroni correction for multiple ...

Table 46. Pairwise comparison of significant KW Living Arrangements for Question 15.

Pairwise Comparisons of What are the living arrangements of the participant?

Sample 1-Sample 2	Test Statistic	Std. Error	Std. Test Statistic	Sig.	Adj. Sig.[a]
Own home (independently)-Own home (supported by family)	-2.566	14.429	-.178	.859	1.000
Own home (independently)-SBC	-70.963	28.341	-2.504	.012	.037
Own home (supported by family)-SBC	-68.397	29.977	-2.282	.023	.068

Each row tests the null hypothesis that the Sample 1 and Sample 2 distributions are the same.
Asymptotic significances (2-sided tests) are displayed. The significance level is .050.

a. Significance values have been adjusted by the Bonferroni correction for multiple ...

Table 47. Pairwise comparison of significant KW Living Arrangements for Question 17.

Pairwise Comparisons of What are the living arrangements of the participant?

Sample 1-Sample 2	Test Statistic	Std. Error	Std. Test Statistic	Sig.	Adj. Sig.[a]
Own home (independently)-Own home (supported by family)	-2.566	14.429	-.178	.859	1.000
Own home (independently)-SBC	-70.963	28.341	-2.504	.012	.037
Own home (supported by family)-SBC	-68.397	29.977	-2.282	.023	.068

Each row tests the null hypothesis that the Sample 1 and Sample 2 distributions are the same.
Asymptotic significances (2-sided tests) are displayed. The significance level is .050.

a. Significance values have been adjusted by the Bonferroni correction for multiple ...

Table 48. Pairwise comparison of significant KW Local Institute for Question 1.

Pairwise Comparisons of Local Institute Country of Origin

Sample 1-Sample 2	Test Statistic	Std. Error	Std. Test Statistic	Sig.	Adj. Sig.[a]
UK-Germany	21.763	22.257	.978	.328	.985
UK-Luxembourg	119.038	18.740	6.352	.000	.000
Germany-Luxembourg	97.275	23.321	4.171	.000	.000

Each row tests the null hypothesis that the Sample 1 and Sample 2 distributions are the same.
Asymptotic significances (2-sided tests) are displayed. The significance level is .050.

a. Significance values have been adjusted by the Bonferroni correction for multiple tests.

Table 49. Pairwise comparison of significant KW Local Institute for Question 2.

Pairwise Comparisons of Local Institute Country of Origin

Sample 1-Sample 2	Test Statistic	Std. Error	Std. Test Statistic	Sig.	Adj. Sig.[a]
UK-Germany	6.002	22.058	.272	.786	1.000
UK-Luxembourg	113.746	18.573	6.124	.000	.000
Germany-Luxembourg	107.744	23.112	4.662	.000	.000

Each row tests the null hypothesis that the Sample 1 and Sample 2 distributions are the same.
Asymptotic significances (2-sided tests) are displayed. The significance level is .050.

a. Significance values have been adjusted by the Bonferroni correction for multiple tests.

Table 50. Pairwise comparison of significant KW Local Institute for Question 4.

Pairwise Comparisons of Local Institute Country of Origin

Sample 1-Sample 2	Test Statistic	Std. Error	Std. Test Statistic	Sig.	Adj. Sig.[a]
UK-Germany	94.481	22.247	4.247	.000	.000
UK-Luxembourg	108.551	18.731	5.795	.000	.000
Germany-Luxembourg	14.070	23.309	.604	.546	1.000

Each row tests the null hypothesis that the Sample 1 and Sample 2 distributions are the same.
Asymptotic significances (2-sided tests) are displayed. The significance level is .050.

a. Significance values have been adjusted by the Bonferroni correction for multiple tests.

Table 51. Pairwise comparison of significant KW Local Institute for Question 5.

Pairwise Comparisons of Local Institute Country of Origin

Sample 1-Sample 2	Test Statistic	Std. Error	Std. Test Statistic	Sig.	Adj. Sig.[a]
Germany-UK	-51.890	22.617	-2.294	.022	.065
Germany-Luxembourg	125.238	23.698	5.285	.000	.000
UK-Luxembourg	73.348	19.043	3.852	.000	.000

Each row tests the null hypothesis that the Sample 1 and Sample 2 distributions are the same.
Asymptotic significances (2-sided tests) are displayed. The significance level is .050.

a. Significance values have been adjusted by the Bonferroni correction for multiple tests.

Table 52. Pairwise comparison of significant KW Local Institute for Question 6.

Pairwise Comparisons of Local Institute Country of Origin

Sample 1-Sample 2	Test Statistic	Std. Error	Std. Test Statistic	Sig.	Adj. Sig.[a]
Germany-UK	-18.782	22.484	-.835	.404	1.000
Germany-Luxembourg	56.243	23.558	2.387	.017	.051
UK-Luxembourg	37.461	18.931	1.979	.048	.144

Each row tests the null hypothesis that the Sample 1 and Sample 2 distributions are the same.
Asymptotic significances (2-sided tests) are displayed. The significance level is .050.

a. Significance values have been adjusted by the Bonferroni correction for multiple tests.

Table 53. Pairwise comparison of significant KW Local Institute for Question 7.

Pairwise Comparisons of Local Institute Country of Origin

Sample 1-Sample 2	Test Statistic	Std. Error	Std. Test Statistic	Sig.	Adj. Sig.[a]
UK-Germany	93.912	22.372	4.198	.000	.000
UK-Luxembourg	102.920	18.836	5.464	.000	.000
Germany-Luxembourg	9.008	23.440	.384	.701	1.000

Each row tests the null hypothesis that the Sample 1 and Sample 2 distributions are the same.
Asymptotic significances (2-sided tests) are displayed. The significance level is .050.

a. Significance values have been adjusted by the Bonferroni correction for multiple tests.

Table 54. Pairwise comparison of significant KW Local Institute for Question 8 .

Pairwise Comparisons of Local Institute Country of Origin

Sample 1-Sample 2	Test Statistic	Std. Error	Std. Test Statistic	Sig.	Adj. Sig.[a]
UK-Germany	57.958	22.531	2.572	.010	.030
UK-Luxembourg	94.994	18.970	5.007	.000	.000
Germany-Luxembourg	37.036	23.607	1.569	.117	.350

Each row tests the null hypothesis that the Sample 1 and Sample 2 distributions are the same.
Asymptotic significances (2-sided tests) are displayed. The significance level is .050.

a. Significance values have been adjusted by the Bonferroni correction for multiple tests.

Table 55. Pairwise comparison of significant KW Local Institute for Question 9

Pairwise Comparisons of Local Institute Country of Origin

Sample 1-Sample 2	Test Statistic	Std. Error	Std. Test Statistic	Sig.	Adj. Sig.[a]
UK-Germany	49.497	22.137	2.236	.025	.076
UK-Luxembourg	70.137	18.639	3.763	.000	.001
Germany-Luxembourg	20.640	23.194	.890	.374	1.000

Each row tests the null hypothesis that the Sample 1 and Sample 2 distributions are the same.
Asymptotic significances (2-sided tests) are displayed. The significance level is .050.

a. Significance values have been adjusted by the Bonferroni correction for multiple tests.

Table 56. Pairwise comparison of significant KW Local Institute for Question 10

Pairwise Comparisons of Local Institute Country of Origin

Sample 1-Sample 2	Test Statistic	Std. Error	Std. Test Statistic	Sig.	Adj. Sig.[a]
UK-Germany	1.834	22.589	.081	.935	1.000
UK-Luxembourg	84.278	19.020	4.431	.000	.000
Germany-Luxembourg	82.445	23.668	3.483	.000	.001

Each row tests the null hypothesis that the Sample 1 and Sample 2 distributions are the same.
Asymptotic significances (2-sided tests) are displayed. The significance level is .050.

a. Significance values have been adjusted by the Bonferroni correction for multiple tests.

Table 57. Pairwise comparison of significant KW Local Institute for Question 11.

Pairwise Comparisons of Local Institute Country of Origin

Sample 1-Sample 2	Test Statistic	Std. Error	Std. Test Statistic	Sig.	Adj. Sig.[a]
UK-Germany	11.778	22.534	.523	.601	1.000
UK-Luxembourg	83.648	18.973	4.409	.000	.000
Germany-Luxembourg	71.870	23.610	3.044	.002	.007

Each row tests the null hypothesis that the Sample 1 and Sample 2 distributions are the same.
Asymptotic significances (2-sided tests) are displayed. The significance level is .050.

a. Significance values have been adjusted by the Bonferroni correction for multiple tests.

Table 58. Pairwise comparison of significant KW Local Institute for Question 12.

Pairwise Comparisons of Local Institute Country of Origin

Sample 1-Sample 2	Test Statistic	Std. Error	Std. Test Statistic	Sig.	Adj. Sig.[a]
UK-Germany	63.105	22.385	2.819	.005	.014
UK-Luxembourg	109.029	18.848	5.785	.000	.000
Germany-Luxembourg	45.924	23.454	1.958	.050	.151

Each row tests the null hypothesis that the Sample 1 and Sample 2 distributions are the same.
Asymptotic significances (2-sided tests) are displayed. The significance level is .050.

a. Significance values have been adjusted by the Bonferroni correction for multiple tests.

Table 59. Pairwise comparison of significant KW Local Institute for Question 13.

Pairwise Comparisons of Local Institute Country of Origin

Sample 1-Sample 2	Test Statistic	Std. Error	Std. Test Statistic	Sig.	Adj. Sig.[a]
UK-Luxembourg	120.078	19.023	6.312	.000	.000
UK-Germany	153.041	22.594	6.774	.000	.000
Luxembourg-Germany	-32.963	23.673	-1.392	.164	.491

Each row tests the null hypothesis that the Sample 1 and Sample 2 distributions are the same.
Asymptotic significances (2-sided tests) are displayed. The significance level is .050.

a. Significance values have been adjusted by the Bonferroni correction for multiple tests.

Table 60. Pairwise comparison of significant KW Local Institute for Question 14.

Pairwise Comparisons of Local Institute Country of Origin

Sample 1-Sample 2	Test Statistic	Std. Error	Std. Test Statistic	Sig.	Adj. Sig.[a]
UK-Germany	70.164	22.520	3.116	.002	.006
UK-Luxembourg	141.840	18.961	7.481	.000	.000
Germany-Luxembourg	71.676	23.595	3.038	.002	.007

Each row tests the null hypothesis that the Sample 1 and Sample 2 distributions are the same.
Asymptotic significances (2-sided tests) are displayed. The significance level is .050.

a. Significance values have been adjusted by the Bonferroni correction for multiple tests.

Table 61. Pairwise comparison of significant KW Local Institute for Question 15.

Pairwise Comparisons of Local Institute Country of Origin

Sample 1-Sample 2	Test Statistic	Std. Error	Std. Test Statistic	Sig.	Adj. Sig.[a]
UK-Germany	9.039	22.549	.401	.689	1.000
UK-Luxembourg	144.470	18.986	7.609	.000	.000
Germany-Luxembourg	135.431	23.626	5.732	.000	.000

Each row tests the null hypothesis that the Sample 1 and Sample 2 distributions are the same.
Asymptotic significances (2-sided tests) are displayed. The significance level is .050.

a. Significance values have been adjusted by the Bonferroni correction for multiple tests.

Table 62. Pairwise comparison of significant KW Local Institute for Question 16.

Pairwise Comparisons of Local Institute Country of Origin

Sample 1-Sample 2	Test Statistic	Std. Error	Std. Test Statistic	Sig.	Adj. Sig.[a]
Germany-UK	-56.909	22.577	-2.521	.012	.035
Germany-Luxembourg	78.865	23.655	3.334	.001	.003
UK-Luxembourg	21.956	19.009	1.155	.248	.744

Each row tests the null hypothesis that the Sample 1 and Sample 2 distributions are the same.
Asymptotic significances (2-sided tests) are displayed. The significance level is .050.

a. Significance values have been adjusted by the Bonferroni correction for multiple tests.

Table 63. Pairwise comparison of significant KW Local Institute for Question 17.

Pairwise Comparisons of Local Institute Country of Origin

Sample 1-Sample 2	Test Statistic	Std. Error	Std. Test Statistic	Sig.	Adj. Sig.[a]
Germany-UK	-35.418	22.636	-1.565	.118	.353
Germany-Luxembourg	107.128	23.717	4.517	.000	.000
UK-Luxembourg	71.710	19.059	3.762	.000	.001

Each row tests the null hypothesis that the Sample 1 and Sample 2 distributions are the same.
Asymptotic significances (2-sided tests) are displayed. The significance level is .050.

a. Significance values have been adjusted by the Bonferroni correction for multiple tests.

Table 64. Pairwise comparison of significant KW Local Institute for Question 18.

Pairwise Comparisons of Local Institute Country of Origin

Sample 1-Sample 2	Test Statistic	Std. Error	Std. Test Statistic	Sig.	Adj. Sig.[a]
UK-Germany	14.288	22.577	.633	.527	1.000
UK-Luxembourg	89.761	19.010	4.722	.000	.000
Germany-Luxembourg	75.473	23.656	3.190	.001	.004

Each row tests the null hypothesis that the Sample 1 and Sample 2 distributions are the same.
Asymptotic significances (2-sided tests) are displayed. The significance level is .050.

a. Significance values have been adjusted by the Bonferroni correction for multiple tests.

Table 65. Pairwise comparison of significant KW Local Institute for Question 19.

Pairwise Comparisons of Local Institute Country of Origin

Sample 1-Sample 2	Test Statistic	Std. Error	Std. Test Statistic	Sig.	Adj. Sig.[a]
UK-Luxembourg	54.551	18.688	2.919	.004	.011
UK-Germany	79.800	22.196	3.595	.000	.001
Luxembourg-Germany	-25.248	23.256	-1.086	.278	.833

Each row tests the null hypothesis that the Sample 1 and Sample 2 distributions are the same.
Asymptotic significances (2-sided tests) are displayed. The significance level is .050.

a. Significance values have been adjusted by the Bonferroni correction for multiple tests.

Table 66. Pairwise comparison of significant KW Local Institute for Question 20.

Pairwise Comparisons of Local Institute Country of Origin

Sample 1-Sample 2	Test Statistic	Std. Error	Std. Test Statistic	Sig.	Adj. Sig.[a]
UK-Luxembourg	135.930	18.935	7.179	.000	.000
UK-Germany	140.596	22.488	6.252	.000	.000
Luxembourg-Germany	-4.666	23.562	-.198	.843	1.000

Each row tests the null hypothesis that the Sample 1 and Sample 2 distributions are the same.
Asymptotic significances (2-sided tests) are displayed. The significance level is .050.

a. Significance values have been adjusted by the Bonferroni correction for multiple tests.

Table 67. Pairwise comparison of significant KW Local Institute for Question 21.

Pairwise Comparisons of Local Institute Country of Origin

Sample 1-Sample 2	Test Statistic	Std. Error	Std. Test Statistic	Sig.	Adj. Sig.[a]
UK-Germany	45.247	22.340	2.025	.043	.128
UK-Luxembourg	60.606	18.810	3.222	.001	.004
Germany-Luxembourg	15.360	23.407	.656	.512	1.000

Each row tests the null hypothesis that the Sample 1 and Sample 2 distributions are the same.
Asymptotic significances (2-sided tests) are displayed. The significance level is .050.

a. Significance values have been adjusted by the Bonferroni correction for multiple tests.

Table 68. Pairwise comparison of significant KW Local Institute for Question 22.

Pairwise Comparisons of Local Institute Country of Origin

Sample 1-Sample 2	Test Statistic	Std. Error	Std. Test Statistic	Sig.	Adj. Sig.[a]
UK-Germany	53.300	22.708	2.347	.019	.057
UK-Luxembourg	154.569	19.120	8.084	.000	.000
Germany-Luxembourg	101.269	23.793	4.256	.000	.000

Each row tests the null hypothesis that the Sample 1 and Sample 2 distributions are the same.
Asymptotic significances (2-sided tests) are displayed. The significance level is .050.

a. Significance values have been adjusted by the Bonferroni correction for multiple tests.

Table 69. Pairwise comparison of significant KW Local Institute for Question 23.

Pairwise Comparisons of Local Institute Country of Origin

Sample 1-Sample 2	Test Statistic	Std. Error	Std. Test Statistic	Sig.	Adj. Sig.[a]
UK-Germany	112.934	22.429	5.035	.000	.000
UK-Luxembourg	140.724	18.885	7.452	.000	.000
Germany-Luxembourg	27.790	23.501	1.183	.237	.711

Each row tests the null hypothesis that the Sample 1 and Sample 2 distributions are the same.
Asymptotic significances (2-sided tests) are displayed. The significance level is .050.

a. Significance values have been adjusted by the Bonferroni correction for multiple tests.

Table 70. Pairwise comparison of significant KW Local Institute for Question 24.

Pairwise Comparisons of Local Institute Country of Origin

Sample 1-Sample 2	Test Statistic	Std. Error	Std. Test Statistic	Sig.	Adj. Sig.[a]
UK-Germany	78.873	22.591	3.491	.000	.001
UK-Luxembourg	130.968	19.021	6.885	.000	.000
Germany-Luxembourg	52.095	23.671	2.201	.028	.083

Each row tests the null hypothesis that the Sample 1 and Sample 2 distributions are the same.
Asymptotic significances (2-sided tests) are displayed. The significance level is .050.

a. Significance values have been adjusted by the Bonferroni correction for multiple tests.

Table 71. Pairwise comparison of significant KW Local Institute for Question 25.

Pairwise Comparisons of Local Institute Country of Origin

Sample 1-Sample 2	Test Statistic	Std. Error	Std. Test Statistic	Sig.	Adj. Sig.[a]
UK-Germany	54.535	22.619	2.411	.016	.048
UK-Luxembourg	124.211	19.044	6.522	.000	.000
Germany-Luxembourg	69.677	23.699	2.940	.003	.010

Each row tests the null hypothesis that the Sample 1 and Sample 2 distributions are the same.
Asymptotic significances (2-sided tests) are displayed. The significance level is .050.

a. Significance values have been adjusted by the Bonferroni correction for multiple tests.

Table 72. Pairwise comparison of significant KW Local Institute for Question 26.

Pairwise Comparisons of Local Institute Country of Origin

Sample 1-Sample 2	Test Statistic	Std. Error	Std. Test Statistic	Sig.	Adj. Sig.[a]
UK-Germany	29.962	22.574	1.327	.184	.553
UK-Luxembourg	89.675	19.007	4.718	.000	.000
Germany-Luxembourg	59.713	23.652	2.525	.012	.035

Each row tests the null hypothesis that the Sample 1 and Sample 2 distributions are the same.
Asymptotic significances (2-sided tests) are displayed. The significance level is .050.

a. Significance values have been adjusted by the Bonferroni correction for multiple tests.

Table 73. Pairwise comparison of significant KW Local Institute for Question 27.

Pairwise Comparisons of Local Institute Country of Origin

Sample 1-Sample 2	Test Statistic	Std. Error	Std. Test Statistic	Sig.	Adj. Sig.[a]
UK-Germany	62.790	22.614	2.777	.005	.016
UK-Luxembourg	105.430	19.040	5.537	.000	.000
Germany-Luxembourg	42.640	23.694	1.800	.072	.216

Each row tests the null hypothesis that the Sample 1 and Sample 2 distributions are the same.
Asymptotic significances (2-sided tests) are displayed. The significance level is .050.

a. Significance values have been adjusted by the Bonferroni correction for multiple tests.
